# Ephrin B Activate Src Family Kinases in Fibroblasts Inducing Stromal Remodeling in Prostate Cancer

**DOI:** 10.3390/cancers14092336

**Published:** 2022-05-09

**Authors:** Mamatha Kakarla, Sathyavathi ChallaSivaKanaka, Mary F. Dufficy, Victoria Gil, Yana Filipovich, Renee Vickman, Susan E. Crawford, Simon W. Hayward, Omar E. Franco

**Affiliations:** Department of Surgery, NorthShore University HealthSystem, Research Institute, 1001 University Place, Chicago, IL 60201, USA; mkakarla@northshore.org (M.K.); schalla@northshore.org (S.C.); mdufficy@northshore.org (M.F.D.); vgil@northshore.org (V.G.); yfilipovich@northshore.org (Y.F.); rvickman@northshore.org (R.V.); crawford7077@gmail.com (S.E.C.); shayward@northshore.org (S.W.H.)

**Keywords:** tumor microenvironment (TME), carcinoma-associated fibroblasts (CAF), stroma, prostate cancer, Ephrins, reverse signaling, Src family kinases, cytokines, Tenascin-C, EFNB1, EFNB2, EFNB3, paracrine signaling

## Abstract

**Simple Summary:**

Carcinoma associated fibroblasts (CAF) play a critical role in the tumor microenvironment (TME) of prostate cancer (PCa). Ephrin receptors (Eph) and ligands (EFN) have been implicated in distinct types of cancers. Alterations on EphB receptors are frequently found in PCa, but the role of ligands (EFNB1, EFNB2, EFNB3) activation in prostate fibroblasts and consequent effects on PCa is not known. We found increased EFNB ligands in fibroblasts isolated from PCa tissues. In this study, we assessed the effects of elevated stromal EFNB ligands on PCa tumor growth. Increased EFNB1 and EFNB3 expression transformed normal fibroblasts into CAF phenotypes through activation of Src family kinases. The secretome of EFNB-expressing CAF increased PCa cell proliferation and promoted TME remodeling. Overall, EFNB activation in CAF may participate in PCa progression via the release of soluble factors that modulate the surrounding tumor environment, which, in turn, promote prostate tumor growth and invasion.

**Abstract:**

Through stromal-epithelial interactions, carcinoma associated fibroblasts (CAF) play a critical role in tumor growth and progression. Activation of erythrophoyetin-producing human hepatocellular (Eph) receptors has been implicated in cancer. Eph receptor interactions with Ephrin ligands lead to bidirectional signals in the recipient and effector cells. The consequences of continuous reverse Ephrin signaling activation in fibroblasts on prostate cancer (PCa) is unknown. When compared to benign prostate fibroblast, CAF displayed higher expression of Ephrin B1, B2, and B3 ligands (EFNB1, EFNB2, and EFNB3). In this study, we found that continuous activation of EFNB1 and EFNB3 in a benign human prostate stromal cell line (BHPrS1) increased the expression of CAF markers and induced a CAF phenotype. BHPrS1^EFNB1^ and BHPrS1^EFNB3^ displayed a pro-tumorigenic secretome with multiple effects on neovascularization, collagen deposition, and cancer cell proliferation, overall increasing tumorigenicity of a premalignant prostate epithelial cell line BPH1 and PCa cell line LNCaP, both in vitro and in vivo. Inhibition of Src family kinases (SFK) in BHPrS1^EFNB1^ and BHPrS1^EFNB3^ suppressed EFNB-induced ɑ-SMA (Alpha-smooth muscle actin) and TN-C (Tenascin-C) in vitro. Our study suggests that acquisition of CAF characteristics via SFK activation in response to increased EFNB ligands could promote carcinogenesis via modulation of TME in PCa.

## 1. Introduction

It is now well established that cancer cells do not exist in isolation, but in a niche known as the tumor microenvironment (TME) where they coexist with a variety of cellular and extracellular components [1,2,3]. The TME comprises a number of non-malignant cell types such as fibroblasts, endothelial cells, immune/inflammatory cells, and extracellular matrix (ECM) components. These cells secrete growth factors, cytokines, and chemokines that can promote tumorigenesis in a paracrine manner [4,5]. Activated fibroblasts, known as carcinoma-associated fibroblasts (CAFs), are the most abundant TME cell population and have been shown to induce cancer by promoting oncogenic signaling pathways and remodeling the TME [5,6,7]. Normal fibroblasts from the benign transition zone (TZ) and the peripheral zone (PZ) have similar transcriptomes compared to fibroblasts from malignant areas in the PZ [8]. In prostate cancer (PCa), CAFs exhibit distinct properties compared to normal fibroblasts. In vitro and in vivo studies have shown that CAFs can enhance tumorigenesis and malignant phenotypes [9,10,11,12]. Crosstalk between cancer cells and CAFs associated with PCa tumor growth and progression occurs either through direct cell-to-cell interaction or paracrine signaling [12,13]. CAFs interact with cancer cells and with other cells in the TME, orchestrating a cascade of events leading to local invasion and metastasis [10]. The CAF secretome has direct effects on cancer cell proliferation and invasion, and indirectly on the TME, allowing formation of new blood vessels [4,5]. Despite a large body of evidence supporting a role for CAFs in prostate tumorigenesis, the exact mechanism(s) by which these cells exert their effects during the initial or later stages of the disease are not completely understood. Therefore, de-coding the mechanisms responsible for CAF activation and crosstalk with cancer cells or other TME components may allow us to target CAFs for potential diagnosis or treatment. 

A family of proteins called Eph’s (erythropoietin-producing hepatocellular receptors), and their membrane bound ligands called Ephrins (erythropoietin-producing hepatocellular receptor interacting proteins), have been shown to be dysregulated and associated with the progression of various cancers [14,15,16]. Eph’s are the largest receptor tyrosine kinase family in the human genome comprising 14 receptors (9 type A and 5 type B) and 8 ligands (5 type A and 3 type B). Eph’s and Ephrins are involved in normal physiological processes such as maintenance of tissue integrity, cell proliferation, angiogenesis, motility, and axon guidance [17,18,19,20]. Eph receptors and Ephrins also regulate pathological conditions such as cancer [21,22,23]. They are expressed in a broad range of human cancer types in both malignant cells and other cells in the TME. Eph receptors and their ligands, Ephrins, interact and trigger bi-directional signaling affecting both Eph expressing cells (forward signaling) and Ephrin-expressing cells (reverse signaling) [15,20,24]. To further add to the complexity of Ephrin reverse signaling, this activation could also be receptor-independent [25,26]. In vitro studies have shown that activation of stromal EFNB2 is important for contact inhibition of locomotion in PCa cells [27]. Expression profiling of human patient samples revealed altered abundances and regulation of Eph receptors and Ephrins to be linked with distinct types of cancers including PCa [28].

Ephrin signaling is complex and has apparent paradoxical effects of both promoting and inhibiting tumorigenesis [15]. For example, the EphB2 receptor has been presented as a putative tumor suppressor gene in PCa [21,28], whereas the EphB4 receptor is known to be both tumor-promoting [22,29] in the presence of its cognate ligand Ephrin B2 (EFNB2) and a tumor suppressor in the absence of EFNB2 [30]. These effects could be influenced by factors such as the target tissue and the action of ligand dependent versus independent signaling in a given context [31]. The literature has been centered on the role of Eph receptors and their ligands, with Ephrins mainly in epithelial cells, and the potential mechanisms that stimulate oncogenic transformation and promotion of tumor invasion and migration. To date, no study has investigated the consequences of receptor-independent continuous reverse Ephrin B signaling activation in the TME in PCa. We observed higher levels of Ephrin B ligands in fibroblasts isolated from PCa patients compared to benign fibroblasts. However, the consequences of high stromal EFNB ligand activation on prostate tumorigenesis is not known. We hypothesized that overexpression of stromal EFNB ligands plays a role in PCa tumorigenesis by inducing CAF activation. Therefore, in this study we sought to determine the downstream molecular consequences of activation of Ephrin ligands in stromal cells. We focused primarily on Ephrin B (EFNB) ligands for their potential role in tumor biology because of their binding affinity to EphB receptors. EphB receptors have been associated with tumor suppression and promotion in different types of cancer, including PCa [32,33,34,35]. However, little is known about the regulation and role of EFNB ligands in PCa TME biology. In this study we report that overexpression of EFNB ligands in benign prostate stromal cells induced a CAF phenotype with pleiotropic intrinsic (fibroblasts) and paracrine (epithelial/cancer cell) effects in vitro and pro-tumorigenic effects in vivo. Our findings indicate that EFNB ligands could have potential clinical value as diagnostic or therapeutic tools.

## 2. Materials and Methods

### 2.1. Real-Time RT-PCR

Upon written informed patient consent and local ethical committee approval (NorthShore University Health System Institutional Review Board-approved collection protocol) de-identified human prostatic tissue samples were obtained from patients with PCa undergoing robotic-assisted laparoscopic prostatectomy (RALP) by the NorthShore Urology Biobank. Prostate fibroblasts isolated from the transition zone (TZ) and peripheral zone (PZ) of patient samples were taken from our laboratory stock. Fibroblasts were cultured in T-75 flasks up to 70–80% confluence, and total RNA was extracted using the RNeasy Mini kit (Qiagen, Germantown, MD, USA) following the manufacturer’s instructions with one modification. To quality control for DNase digestion of RNA, a 5 PRIME RNase-free DNase kit was used prior to transferring RNA solution to the column. Isolated RNA was reverse-transcribed into cDNA using an I Script cDNA synthesis kit (Bio-Rad, Hercules, CA, USA) following the manufacturer’s protocol. Human EFNB1, EFNB2, and EFNB3 (VHPS-2876, VHPS-2877, VHPS-2878, Real Time Primers, Elkins Park, PA, USA) mRNAs were amplified using synthetic oligomers as probes and the reactions were performed in triplicates with iTaq universal SYBR Green supermix (Bio-Rad) on QuantStudio 7 Flex real-time PCR system (Applied Biosystems, Waltham, MA, USA) including no template controls.

### 2.2. Cell Lines and Reagents

Benign human prostate stromal cells (BHPrS1) were obtained from our stock and maintained in RPMI-1640 medium (Gibco, Cat# 22400-089, Waltham, MA, USA) supplemented with 10% calf serum (HyClone, Cat# SH30087.03, Logan, UT, USA) [9]. In this study, we used two prostate epithelial cell lines: pre-malignant human prostate cell line BPH1 (our own stocks), and lymph node metastatic LNCaP (ATCC CRL-1740). BPH1 and LNCaP cells were maintained in RPMI-1640 (Gibco, 22400-089) with 10% fetal bovine serum (R&D Systems, Minneapolis, MN, USA). In addition, cell culture media for both stromal and epithelial cells were supplemented with 1% penicillin/streptomycin (Life technologies, Carlsbad, CA, USA). Cells were cultured at 37 °C in a humidified incubator under 5% CO_2_. Cell culture media was changed every 3–4 days depending on cell density. For routine passage, cells were harvested with 0.25% trypsin-EDTA and split at a ratio of 1:3 when they were 80–90% confluent.

### 2.3. Plasmid Constructs and Stable Cell Line Generation

Clones for Ephrin ligands-EFNB1 (Ephrin-B1), EFNB2 (Ephrin-B2), and EFNB3 (Ephrin-B3) were obtained from the DNASU plasmid repository [36]. EFNB1 in pDONR221 (HsCD00043900), EFNB2 in pENTR223 (HsCD00289024), and EFNB3 in pENTR223 (HsCD00515523) were cloned into pLenti CMV Blast DEST (706-1) (Plasmid# 17451, Addgene, Watertown, MA, USA) to generate pLenti CMV Blast vectors with overexpression of either EFNB1 or EFNB2 or EFNB3 using the Gateway LR reaction (Invitrogen, Waltham, MA, USA). pLenti CMV Blast DEST (706-1) was a gift from Eric Campeau & Paul Kaufman (Addgene plasmid# 17451; http://n2t.net/addgene:17451; RRID:Addgene_17451) [37]. Ephrin ligands-overexpressed lentiviruses were produced by stable transfection of 4.3 µg pLenti expression vector and 1 µg/uL of ViralPower Lentiviral Packaging Mix (Invitrogen, Waltham, MA, USA) into 293FT cells in a 10 cm dish with Lipofectamine 3000 reagent (Invitrogen) according to the manufacturer’s instructions. Viral supernatants were collected 48 h post-transfection and passed through a 0.2 µm filter and stored at −80 °C. Cells were transduced in the presence of 4 µg/mL of polybrene (Sigma-Aldrich, St. Louis, MO, USA) for 12 h followed by 2 µg/mL blasticidin (InvivoGen, San Diego, CA, USA) selection for one week. Under selection with blasticidin, cell media was changed every two days. Upon selection with blasticidin, stable cell lines (BHPrS1^EV^/BHPrS1^EFNB1^/BHPrS1^EFNB2^/BHPrS1^EFNB3^) were generated and passaged for subsequent use.

### 2.4. Western Blot Analysis

Cells (100 k per well) were plated in a 6-well plate and cultured for 48 h before being lysed with RIPA (Radio immunoprecipitation assay) lysis buffer (Thermo Scientific, Waltham, MA, USA) supplemented with phosphatase inhibitors (Sigma-Aldrich, St. Louis, MO, USA) and protease inhibitors (Thermo Scientific, Waltham, MA, USA). Protein concentrations were determined using the Pierce BCA Protein Assay kit (Thermofisher, Waltham, MA, USA) and 30 µg of total protein was resolved on 10–15% sodium dodecyl sulfate polyacrylamide gel electrophoresis, transferred to nitrocellulose membrane (Bio-Rad, Hercules, CA, USA) via Trans-blot Turbo Transfer system (Bio-Rad, Hercules, CA, USA) and probed. Anti-Alpha smooth muscle actin (α-SMA, Cat# A5228, Sigma-Aldrich, St. Louis, MO, USA), anti-Tenascin-C (TN-C, Cat# Ab19011, Abcam, Waltham, MA, USA), and anti-Vimentin (Cat# Ab8069, Abcam, Cambridge, UK) were used at 1:1000 dilutions. Anti-EFNB1 (Cat# 34-3500, Invitrogen, Waltham, MA, USA), anti EFNB2 (Cat# Ab131536, Abcam, Cambridge, UK), and anti-EFNB3 (Cat# AB53063, Abcam, Cambridge, UK) were used at 1:500 dilutions, and anti-β-actin (Cat# SC-47778, Santa Cruz Biotechnology, Dallas, TX, USA) was used at 1:5000 dilutions to probe for their respective proteins. Appropriate peroxidase conjugated secondary antibodies were used to label the proteins. The proteins were detected by an enhanced chemiluminescence detection kit (Bio-Rad, Hercules, CA, USA) on a ChemiDoc imaging system (Bio-Rad, Hercules, CA, USA) and densitometric analysis was performed using an image lab software analysis tool (v6.1, Bio-Rad, Hercules, CA, USA). The results were corrected for protein loading by normalization for β-actin expression. The Supplementary Materials contain all the Western blot figures (Figures S8–S12).

### 2.5. Cell Proliferation Assay

Engineered BHPrS1 cells were cultured to 80–90% confluence in complete medium and then replaced with 0.1%BSA-RPMI medium. The cells were then cultured for 72 h and culture supernatant (conditioned media) was collected and stored at −80 °C for downstream analysis. BPH1 and LNCaP cells were seeded in 96 well plates in complete medium at cell densities of 3000 and 8000 cell per well, respectively. Once BPH1 cells became adherent, complete medium was replaced with 0.1% BSA-RPMI medium. After the cells were incubated overnight, the medium was changed to conditioned media (CM) collected from each of the engineered BHPrS1 cell lines (Day 0). For LNCaP cells, conditioned medium and complete medium were used at a 1:1 ratio. The cells were incubated and conditioned media were replaced every 48 h. After 5 days of incubating with conditioned media, cells were fixed with 4% paraformaldehyde (PFA) for 15 min and stained with 0.1% crystal violet stain for 20 min. After thorough washing with tap water, plates were air-dried and cells were solubilized with 10% acetic acid for 20 min. Cell proliferation was evaluated by measuring the absorbance of each well at 590 nm with a SpectraMax Plus 384 microplate reader (Molecular Devices, San Jose, CA, USA).

### 2.6. Migration Assay (Wound Healing Assay or WHA)

Cell migration was determined using a wound healing assay. BPH1 cells were seeded in 12 well plates at a cell density of 100,000 cells per well in complete medium. After 24 h, when cell confluency reached 70–80%, medium was replaced with RPMI containing 0.1% BSA and allowed to incubate overnight. Medium was then replaced with conditioned media from engineered BHPrS1 cells. A sterile 200 µL pipette tip was used to gently scratch a cell-free gap in the shape of a plus sign. The scratch was imaged immediately (Day 0) and then imaged again after 24 h (Day 1) with an EVOS Fl inverted microscope at 10× magnification. Scratch closure was quantified by measuring the cell free area using Image J software and calculating percent of area reduction between Day 0 and Day 1.

### 2.7. Cytokine Array

The expression of 105 human secreted cytokines in each of the engineered BHPrS1 cells were evaluated using an HXL human cytokine antibody array kit (Cat# ARY022B, R&D Systems, Minneapolis, MN, USA). Human XL Cytokine Arrays were incubated overnight at 4 °C with 500 µL conditioned media, and the procedure was performed according to the manufacturer’s instructions. Following incubation with a detection antibody cocktail, antibody conjugation, and recommended washes, the immunoblots on the membrane were developed with the Chemiluminescent Substrate Reagent Kit (Bio-Rad, Hercules, CA, USA). Signals on each array were detected using ChemiDoc Imaging software (Bio-Rad, Hercules, CA, USA) and signal intensity was quantified using the Fiji plugin in ImageJ software (Version 1.53o) [38]. The mean signal intensities were subtracted from the median background intensities for background correction. Up (≥1.5-fold) or downregulation (≤0.5-fold) in cytokine secretion were considered significant (*p* < 0.01) in proteins showing a signal density value >200 pixels.

### 2.8. Human Phospho-Kinase Array

Human phosphokinase antibody array (ARY003C, R&D Systems, Minneapolis, MN, USA) was used to detect the expression of 43 kinase phosphorylation sites on proteins isolated from BHPrS1^EV^/BHPrS1^EFNB1^/BHPrS1^EFNB2^/BHPrS1^EFNB3^ cells. A total of 400 µg of cell lysate per sample was incubated with antibody array membranes in a multiwell dish overnight and analyzed following the manufacturer’s protocol. Signals were detected using ChemiDoc Imaging software (Bio-Rad, Hercules, CA, USA) and densitometric analysis was carried out using the Fiji plugin in ImageJ software [38].

### 2.9. Animal Studies

Animal studies were approved by the Institutional Animal Care and Use Committee (IACUC) of Northshore University Health System. All mice in this study were maintained under constant environmental conditions in the Animal Research Facility of NorthShore University Health system with free access to food and water. A total of 250,000 epithelial cells (BPH1/LNCaP) were combined with 100,000 stromal cells (BHPrS1^EV^/BHPrS1^EFNB1^/BHPrS1^EFNB2^/BHPrS1^EFNB3^) in neutralized rat tail collagen to make tissue recombinants and incubated at 37 °C overnight. The recombinants were grafted under the kidney capsules of intact male CB17Icr/Hsd-SCID mice (Envigo, Denver, PA, USA) and supplemented with 5-mg testosterone via subcutaneously implanted testosterone pellets [9]. One or two grafts were placed under the renal capsule of each kidney. Animals were monitored thoroughly until euthanized.

### 2.10. Xenograft Processing and Staining

Mice were sacrificed using a carbon dioxide chamber for necropsy. BPH1 grafts grew for 8 weeks, while LNCaP grafts grew for 6 weeks, before euthanasia. Kidneys were harvested, measured, photographed, and fixed in formalin. Imaged kidneys were used to measure tumor growth on the kidney using the Fiji plugin in ImageJ software [38]. Briefly, the grafts were imaged, and tumor length, width and height were quantified in image J software. Tumor volume was calculated using an ellipsoid formula as previously described [9]. Kidneys were cut into halves, processed, and paraffin embedded. The sections were cut at 4 µm for Hematoxylin and Eosin (H&E) staining and immunohistochemistry (IHC). The sections were deparaffinized with xylene for 3 min (3×), washed in 100% alcohol for 3 min, 90% alcohol for 3 min and 70% alcohol for 1 min. In the next step, we performed antigen retrieval using an antigen unmasking solution (Vector Laboratories, Burlingame, CA, USA) for Ki-67 staining (20 min boiling at microwave power level II followed by 1-h cooling) and Proteinase K (20 µg/mL) for TN-C staining (heated at 37 °C for 5 min) followed by rinsing in PBS 5 min (3×). The Vectastain Elite kit (Vector Laboratories, Burlingame, CA, USA) was used following manufacturer’s instructions using Ki67 antibody (Cat# ab238020, Abcam, Cambridge, UK) or TN-C antibody (T2551, Sigma-Aldrich, St. Louis, MO, USA). The incubation of primary antibodies was performed in a wet chamber at 4 °C overnight. Collagen deposition was stained using a picrosirius red stain kit (Polysciences, Warrington, PA, USA) following the manufacturer’s instructions.

## 3. Results

### 3.1. Increased Expression of EFNB Ligands in Fibroblasts from the Peripheral Zone of Human Prostate

**Figure 1:**
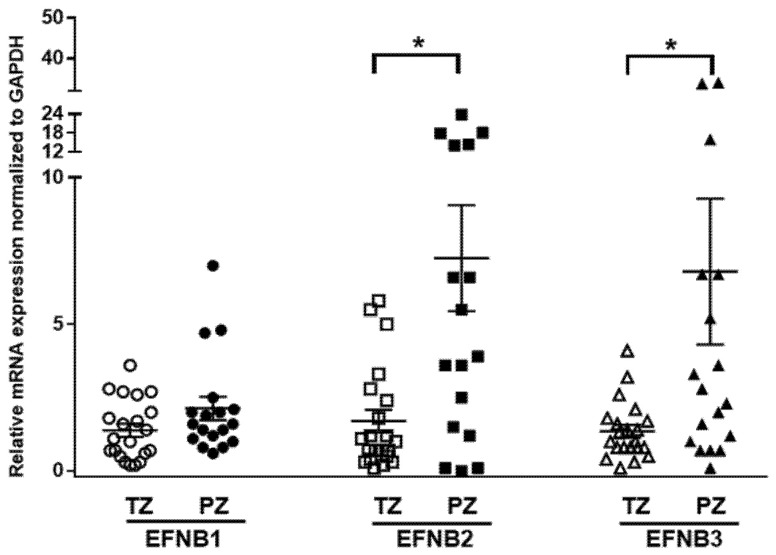
Differential expression of Ephrin B ligands in prostate fibroblasts isolated from the transition zone (TZ) and peripheral zone (PZ) tissues of patients with prostate cancer. Relative expression of Ephrin B1 (EFNB1), Ephrin B2 (EFNB2), and Ephrin B3 (EFNB3) mRNA in fibroblasts isolated from TZ (open shapes) and PZ (solid shapes). Data are shown as dot plots with mean values. Unpaired *t*-test was used for comparison of Ephrin B ligands expression between TZ (*n* = 21) and PZ (*n* = 18), * *p* < 0.05.

Previous studies assessed the expression of Ephrin receptors and ligands in PCa cells and epithelial compartments but not in the tumor stroma [21,39,40]. We determined the mRNA expressions of Ephrin B ligands in human prostate fibroblasts isolated (*n* = 39) from benign (TZ) and malignant peripheral tissues (PZ) using quantitative RT-PCR. Overall, expression of EFNB ligand transcripts was higher in PZ compared to TZ (*p* = 0.056, Figure 1). The mRNA expression of EFNB2 was significantly higher in PZ compared to TZ (7.26 ± 1.8, *n* = 17 vs. 1.7 ± 0.39, *n* = 21; *p* = 0.0011). Similarly, mRNA levels of EFNB3 were significantly higher in PZ compared to TZ (6.8 ± 2.485, *n* = 18 vs. 1.36 ± 0.21, *n* = 21; *p* = 0.0216). Stromal EFNB1 expression was elevated in fibroblasts from PZ (2.14 ± 0.39, *n* = 18), but did not reach significance when compared to TZ (1.39 ± 0.22, *n* = 21). Next, to determine whether the expression of these EFNB paralogs correlate to each other, we evaluated the relationship between EFNB ligands at mRNA level in primary prostate fibroblasts. In primary prostate fibroblasts, EFNB2 was found to have significant positive relationships with EFNB1 (Pearson correlation coefficient = 0.480, *p* = 0.002) and EFNB3 (Pearson correlation coefficient = 0.332, *p* = 0.041). These results suggest that the increased stromal EFNB ligands observed in fibroblasts associated with cancer could have a role in PCa tumorigenesis. 

### 3.2. Overexpression of Stromal EFNB1 and EFNB3 Induce the Expression of CAF Markers

**Figure 2:**
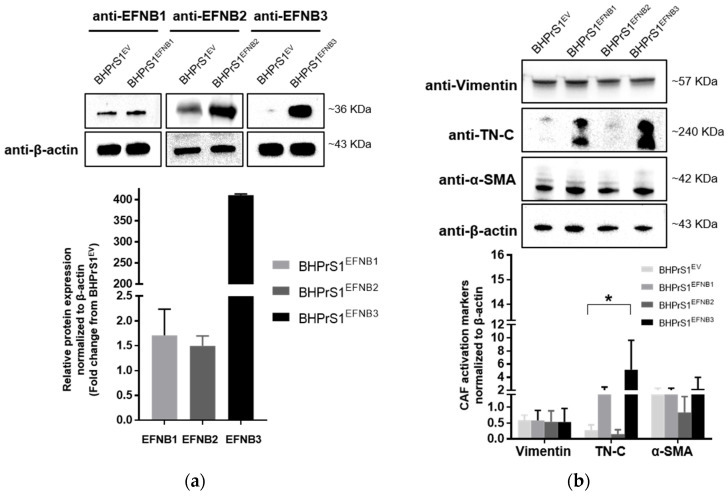
Overexpression of Ephrin B ligands in normal prostate fibroblasts induce the activation of cancer associated fibroblasts (CAF) markers. (**a**) Expression of EFNB1, EFNB2, and EFNB3 ligand proteins in lentivirus transduced BHPrS1 cells was validated by Western blot. The bands were quantified and normalized to β-actin and presented as fold change in protein expression compared to BHPrS1^EV^ (*n* = 3 independent experiments). (**b**) The protein levels of Vimentin and putative CAF markers, Tenascin-C (TN-C), and alpha-smooth muscle actin (α-SMA) were evaluated in Ephrin-generated cell lines (BHPrS1^EFNB1^, BHPrS1^EFNB2^, BHPrS1^EFNB3^) by Western blot. The bands were quantified and normalized to β-actin and presented as the mean (* *p* < 0.05, one-way ANOVA, *n* = 3 independent experiments).

Whether increased EFNB ligands observed in human fibroblasts regulate the expression of putative CAF markers has not been previously studied. Here, a human benign prostate stromal cell line, BHPrS1, was engineered using a lentiviral system to express individual EFNBs. Western blot analysis demonstrated higher levels of BHPrS1^EFNB1^, BHPrS1^EFNB2^, and BHPrS1^EFNB3^, respectively, compared to BHPrS1^EV^ (Figure 2a). It is noteworthy that BHPrS1 cells had high basal levels of EFNB1. Although forced expression significantly induced EFNB1 levels, the fold changes were minimal compared to those induced in BHPrS1^EFNB2^ and BHPrS1^EFNB3^. Expression of previously proposed CAF markers was examined in EFNB engineered cell lines. As shown in Figure 2b, compared to BHPrS1^EV^, TN-C expression increased in BHPrS1^EFNB1^ and BHPrS1^EFNB3^ cell lines. There was no significant increase in α-SMA and vimentin expression between the cell lines. It has been previously proposed that immunophenotypic criteria of myofibroblasts include increased expression of α-SMA in vimentin-positive fibroblasts. The ratio of α-SMA/vimentin in BHPrS1^EFNB3^ was higher compared to control cells (4.2 vs. 2.3), whereas, the ratio of α-SMA/vimentin in BHPrS1^EFNB2^ was lower compared to BHPrS1^EV^ (1.5 vs. 2.3). In contrast to EFNB1 and EFNB3 effects, increased EFNB2 expression in the BHPrS1 cell line did not change the levels of any myofibroblast markers tested, indicating that this ligand may not participate or have a role in myofibroblast/CAF activation. These results show that both BHPrS1^EFNB1^ and BHPrS1^EFNB3^ were phenotypically activated BHPrS1 from its normal basal fibroblast status to a myofibroblast/CAF upon increasing the expression of EFNB1 and EFNB3.

### 3.3. EFNB Ligands Promote Prostate Epithelial Cell Proliferation and Migration in a Paracrine Manner

**Figure 3:**
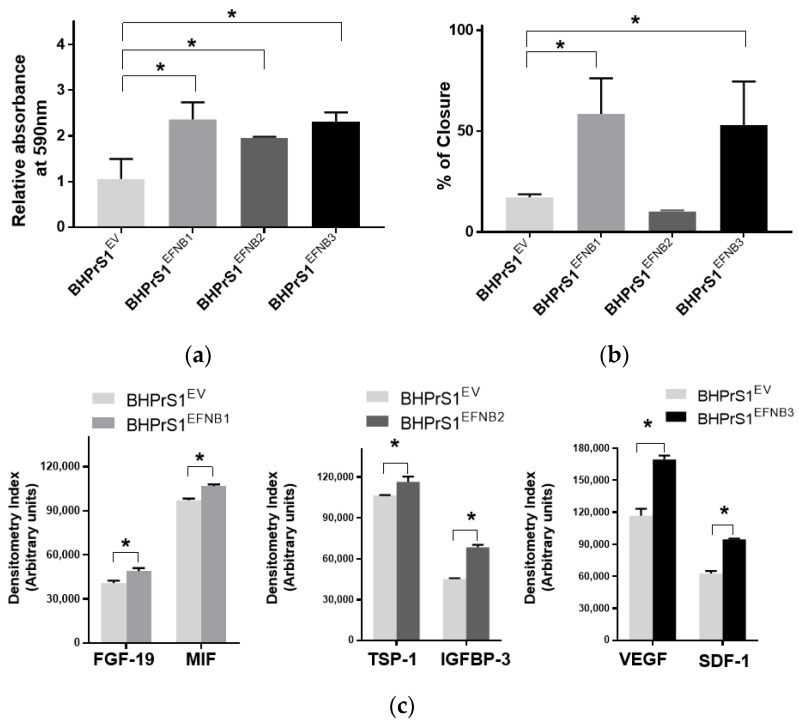
Paracrine signals from Ephrin B-engineered BHPrS1 cells regulate BPH1 cell proliferation and migration. (**a**) BPH1 cell proliferation cultured in the presence of Ephrin B expressing BHPrS1 conditioned media (BHPrS1^EFNB1^, BHPrS1^EFNB2^, BHPrS1^EFNB3^) and compared with control cells (BHPrS1^EV^). One-way ANOVA with Dunnett multiple comparison tests indicate that BPH1 exposed to conditioned media from EFNB ligands overexpressing fibroblasts increased in vitro proliferation (*n* = 5 independent experiments, * *p* < 0.05). (**b**) Quantification of percent scratch closure. Data are presented as the mean ± SEM of three independent experiments (* *p* < 0.05, one-way ANOVA). BPH1 cell closure did not change in the presence of BHPrS1^EFNB2^ conditioned medium compared to control (BHPrS1^EV^). (**c**) Selected cytokines secreted from each of the engineered BHPrS1 cells that are significantly different from control (BHPrS1^EV^) (* *p* < 0.05). FGF-19: Fibroblast growth factor 19; MIF: Macrophage migration inhibitory factor; TSP1: Thrombospondin 1; IGFBP3: Insulin like growth factor binding protein 3; VEGF: Vascular endothelial growth factor; SDF-1: Stromal cell-derived factor-1.

Our previous studies showed that modification of genes associated with the CAF phenotype can alter the secretome in BHPrS1. Secretions from these cells and CAFs are known to promote epithelial cell proliferation both in vitro and in vivo [13]. In order to determine whether engineered EFNB ligand in BHPrS1 cells modulate their paracrine effects on prostate cancer cells, we harvested conditioned media (CM) from cultured fibroblasts and then treated two prostate epithelial cell lines representing premalignant (BPH1) and malignant (LNCaP) states in PCa for 5 days. We saw diverse effects of fibroblast secretions on BPH1 cells (Figure 3a) and LNCaP cells (Figure S1). Secretions from the overexpression of all three EFNB ligands in BHPrS1 cells stimulated proliferation of BPH1 cells in vitro (Figure 3a). Compared to BHPrS1^EV^, BPH1 cell proliferation increased in the presence of conditioned media from BHPrS1^EFNB1^ (*n* = 5, *p* = 0.005), BHPrS1^EFNB2^ (*n* = 5, *p* = 0.048), and BHPrS1^EFNB3^ (*n* = 5, *p* = 0.006). In order to determine if CM contains factors that modulate BPH1 migration, we performed a wound healing assay (WHA) on BPH1 cells (Figure 3b). After 24 h, CM collected from BHPrS1^EFNB1^ and BHPrS1^EFNB3^ closed the gap (58.5 ± 17.6 and 52.9 ± 21.5 respectively) to a significantly greater extent (*n* = 5, *p* < 0.05) compared to BHPrS1^EV^ (17.2 ± 1.5). There was no change in gap closure between control and BHPrS1^EFNB2^ (10.1 ± 0.5, *p* = 0.76, *n* = 5). These results suggest that soluble factors in CM from BHPrS1^EFNB1^ and BHPrS1^EFNB3^, but not BHPrS1^EFNB2^, promote BPH1 cell migration. In contrast to BPH1, LNCaP growth in response to EFNB1-expressing BHPrS1 cells CM had no significant changes. BHPrS1^EFNB2^ CM decreased LNCaP proliferation but the change was not significant (Figure S1, *n* = 5, *p* > 0.05). Interestingly, BHPrS1^EFNB3^ CM showed a trend of increased LNCaP cells proliferation compared to controls. These results suggest that soluble factors from stromal cells expressing EFNB ligands may have different pro (or anti) proliferative/migratory roles in a disease stage-specific manner.

### 3.4. Increased EFNB1 and EFNB3 Expression in BHPrS1 Cells Induce the Secretion of Pro-Tumorigenic and Pro-Angiogenic Factors

To better understand the potential paracrine factor(s) responsible for the in vitro effects observed on BPH1 and LNCaP cells, next, we analyzed the secretion of a panel of cytokines and chemokines from fibroblasts expressing different EFNB ligands. The conditioned medium from each of the engineered BHPrS1 cell lines showed differential secretions in each of the cell lines. Out of 102 cytokines quantified, we identified 41 cytokines in BHPrS1^EFNB1^, 42 in BHPrS1^EFNB2^, and 49 in BHPrS1^EFNB3^ that differentially expressed compared to BHPrS1^EV^ (Figure S2a). Overall, the profile of BHPrS1^EFNB1^ secreted factors included pro-inflammatory and mitogenic genes such as fibroblast growth factor-19 (FGF-19) and Macrophage Migration Inhibitory Factor (MIF) (Figure 3c). Antiangiogenic Thrombospondin-1 (TSP-1), and anti-proliferative Insulin Like Growth Factor Binding Protein 3 (IGFBP-3) were enriched in the BHPrS1^EFNB2^ secreteome (Figure 3c). Overexpression of EFNB3 ligand in BHPrS1 resulted in the secretion of a number of factors with pro-inflammatory, neo-angiogenic, and mitogenic effects such as vascular endothelial cell growth factor (VEGF), stromal-cell derived factor-1 (SDF-1), interleukin-10 (IL-10), interleukin-11 (IL-11), and growth-differentiation factor-15 (GDF-15) (Figure 3c). Tumor angiogenesis in the TME promotes not only cancer cell proliferation, but also has roles during invasion and metastasis. Cytokines such as VEGF, a pro-angiogenic factor, promote tumor growth, whereas TSP-1, an anti-angiogenic factor, reduces cell proliferation. Although the secretions of VEGF and TSP-1 were differentially expressed in BHPrS1^EFNB2^ and BHPrS1^EFNB3^ compared to BHPrS1^EV^, their changes suggest opposing functional directions. When we evaluated the ratio of TSP-1 secretions to VEGF secretions in BHPrS1^EFNB2^ and BHPrS1^EFNB3^ cells, the ratio in BHPrS1^EFNB2^ was greater (1.7) than in BHPrS1^EFNB3^ cells (0.7) (Figure S2b). These results suggest that each EFNB ligand might exert its paracrine action through diverse effects directly (on cancer cells) or indirectly (via TME) during prostate carcinogenesis. To better understand the biological effects of these EFNB-expressing stromal cells, we tested their role in an in vivo assay of stromal-epithelial interactions in PCa.

### 3.5. Higher Levels of Stromal EFNB1 and EFNB3 Promote PCa Tumorigenicity In Vivo

**Figure 4:**
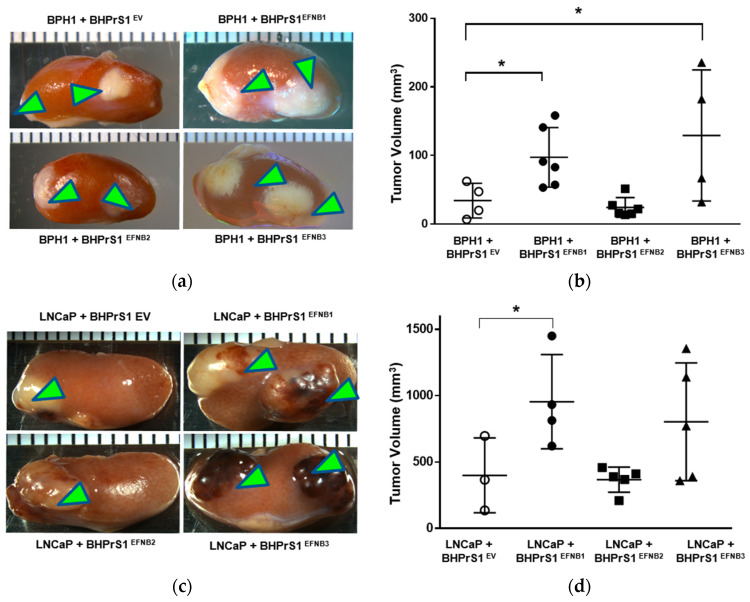
Overexpression of Ephrin B (EFNB) ligands in normal prostate fibroblasts induces in vivo prostate cancer tumorigenicity in SCID xenografts. (**a**) Gross morphology of grafts composed of BPH1 cells with EFNB ligands-overexpressing BHPrS1 cells (BHPrS1^EV^, BHPrS1^EFNB1^, BHPrS1^EFNB2^, BHPrS1^EFNB3^). (**b**) The size of BPH1 tumors in each sample are shown as dot plots with mean ± SEM (* *p* < 0.05, *n* = 4 or 6, one-way ANOVA). (**c**) Gross morphology of grafts composed of LNCaP cells with EFNB ligands overexpressing BHPrS1 cells (BHPrS1^EV^, BHPrS1^EFNB1^, BHPrS1^EFNB2^, BHPrS1^EFNB3^). (**d**) The size of LNCaP tumors in each sample are shown as dot plots with mean ± SEM (* *p* < 0.05, *n* = 3 or 5, one-way ANOVA). Green arrowheads point the tumor on kidney.

To better understand the potential in vivo significance of the above observations, we investigated the effect of stromal EFNB ligand overexpression on PCa tumor growth and/or invasion using a renal capsule xenograft mouse model of PCa in SCID mice. We made recombinants of BPH1 or LNCaP cells with different EFNB-engineered BHPrS1 cells and grafted under the kidney capsules of adult male SCID mice (Figure 4a,c). BPH1 cells are normally non-tumorigenic. However, CAFs, but not normal prostate fibroblasts (NPF), induced a malignant transformation and caused them to grow and invade neighboring renal tissues [12]. The BPH1/CAF model has been shown to recapitulate the initial stages of PCa. The in vivo tumorigenicity of BPH1 was significantly increased in the presence of BHPrS1^EFNB1^ and BHPrS1^EFNB3^ compared to BHPrS1^EV^ (Figure 4b). Despite the pro-proliferative effects in vitro (Figure 3a), we did not see any change in BPH1 tumorigenicity in the presence of BHPrS1^EFNB2^ compared to BHPrS1^EV^ (*p* = 0.73) (Figure 4b). In the presence of activated fibroblasts, the tumorigenicity and invasion of PCa cells increased in vivo [13,41,42]. We also looked at whether enhanced stromal EFNB ligands affect the PCa aggressive cell line LNCaP in vivo tumorigenicity (Figure 4c). There was a significant increase in tumor size of LNCaP in the presence of BHPrS1^EFNB1^ compared to controls (Figure 4d). There was enhanced tumor growth in the presence of BHPrS1^EFNB3^, but the changes did not reach significance (*p* = 0.11). Increased stromal EFNB2 did not accelerate tumor growth in LNCaPs, similar to BPH1 cells (Figure 4d). We and others have previously shown that CAFs induce invasiveness of tumors. Although not significant, we saw increased invasiveness of LNCaP tumors in the presence of BHPrS1^EFNB1^ (2.3) and BHPrS1^EFNB3^ (2.8) compared to BHPrS1^EV^ (1.6) (data not shown). There was no significant change in invasiveness upon increased expression of EFNB2 (1.7) in fibroblasts (data not shown). These results indicate enhanced PCa tumorigenicity in response to increased stromal EFNB1 and EFNB3 ligands. Next, we evaluated how the TME responded to higher stromal EFNB ligands by analyzing in vivo xenograft tissues.

### 3.6. Elevated Stromal Ephrin B Ligands Are Associated with TME Remodeling In Vivo

**Figure 5:**
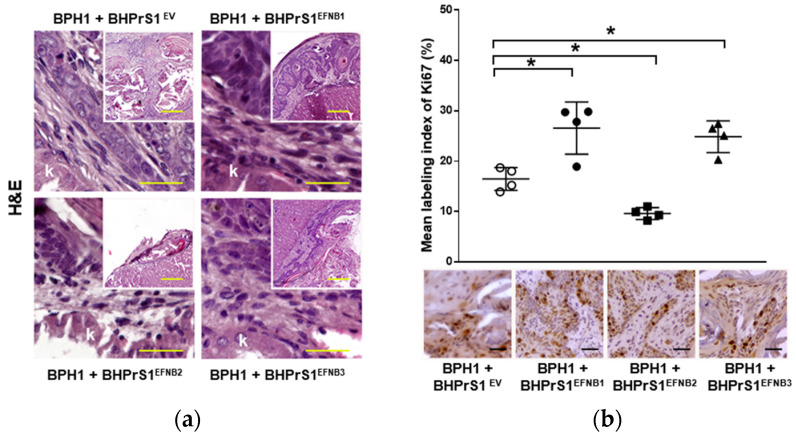

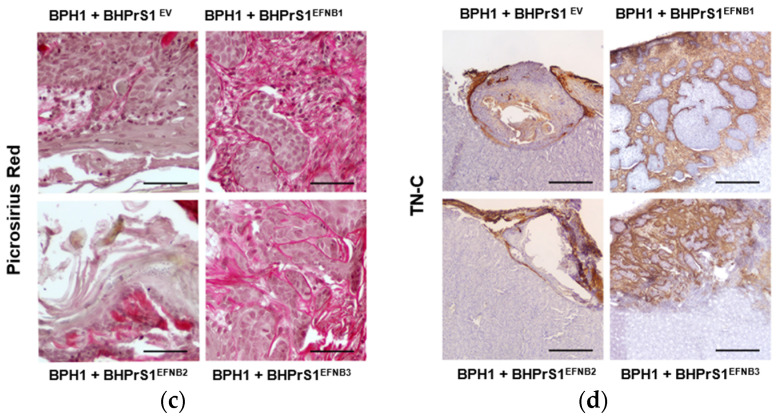
Increased stromal EFNB1 and EFNB3 is associated with enhanced proliferation of BPH1 cells and stromal remodeling in vivo. (**a**) Hematoxylin and eosin (H&E) stained sections of the tumors resulting from grafts of BPH1 with engineered BHPrS1 cell lines. Grafts of BPH1 with BHPrS1^EFNB1^ and BHPrS1^EFNB3^ show pronounced inflammatory infiltrates compared to BHPrS1^EV^. (**b**) Immunohistochemical (IHC) staining showing pronounced Ki67 expression in BPH1 tumors with BHPrS1^EFNB1^ and BHPrS1^EFNB3^ and reduced Ki67 expression in BHPrS1^EFNB2^ compared to BHPrS1^EV^. Dot plot showing Ki67 expression quantification (* *p* < 0.05, one-way ANOVA). (**c**) Higher collagen deposition in BPH1 tumor grafts with BHPrS1^EFNB1^ and BHPrS1^EFNB3^ compared to BHPrS1^EV^ as shown by picrosirius red staining. (**d**) ECM remodeling marker Tenascin-C (TN-C) is highly expressed in tumor grafts of BPH1 with BHPrS1^EFNB1^ and BHPrS1^EFNB3^ compared to BHPrS1^EV^. Grafts of BPH1 with BHPrS1^EFNB2^ and BHPrS1^EV^ has relatively lower expressions of TN-C. k in the figure represent Kidney. Scale bars in yellow and black lines in the figures represent that pictures were taken at the same magnification.

Histopathological evaluation of xenografts containing BPH1 cells show the typical adenosquamous phenotype induced in these cells by fibroblasts. In addition to increased epithelium, both BHPrS1^EFNB1^ and BHPrS1^EFNB3^ tumors were composed of a significant amount of stroma between cancer cells. However, in BHPrS1^EV^ and BHPrS1^EFNB2^ grafts, the stroma tended to encapsulate the epithelium (Figure 5a). BHPrS1^EFNB1^ displayed “collective invasion” while BHPrS1^EFNB3^ induced a more focal invasion on the surface of the kidney. Evaluation of Ki67 labeling index aligned with the assessment of tumor growth, with BHPrS1^EFNB1^ and BHPrS1^EFNB3^ proliferation significantly higher than that of BHPrS1^EV^ and BHPrS1^EFNB2^ (Figure 5b). Notably, BHPrS1^EFNB2^ had a significantly decreased proliferation index compared to control cells. To better understand the stromal changes in response to each EFNB ligand, we performed picrosirius red staining to assess collagen deposition in the grafts. Compared to BHPrS1^EV^ and BHPrS1^EFNB2^, collagen accumulation (red staining under light microscopy) was significantly enhanced in BHPrS1^EFNB1^ and BHPrS1^EFNB3^ tumors (Figure 5c) suggesting the induction of stromal remodeling. TN-C is rarely expressed in normal benign tissues; however, in pathologic tissues such as during inflammation, wound healing, and cancer, TN-C is strongly up-regulated and participates in the fibrotic changes associated with these conditions. Evaluation of TN-C expression in the xenografts mirrored our in vitro observations (Figure 2b) with tumors containing BHPrS1^EFNB1^ and BHPrS1^EFNB3^ cells showing strong TN-C expression, occupying almost 100% of the tumor stroma (Figure 5d). LNCaP cells usually show a strong response to stromal cues in this recombination model, yielding subsequent tumor progression. Unlike BPH1 cells, LNCaP were more invasive in the presence of BHPrS1^EFNB1^ and BHPrS1^EFNB3^ compared to control BHPrS1^EV^ cells (Figure S3). When LNCaP cells were combined with BHPrS1^EFNB2^, cancer cells were less invasive (Figure S3a). Evaluation of Ki67 in these tumors aligned with tumor size (Figure S3b). Stromal changes (Trichrome or Picrosirius Red and TN-C) were similar to those seen in BPH1 tumors (Figure S3c,d). These results suggest that in addition to the epithelial proliferation and invasion induced by stromal Ephrins, there is a significant remodeling of the TME associated with collagen deposition and TN-C expression, which are both factors known to be involved in cancer cell survival, invasion, and tumor progression.

### 3.7. EFNB1 and EFNB3 Activate SFK in Prostate Fibroblasts

**Figure 6:**
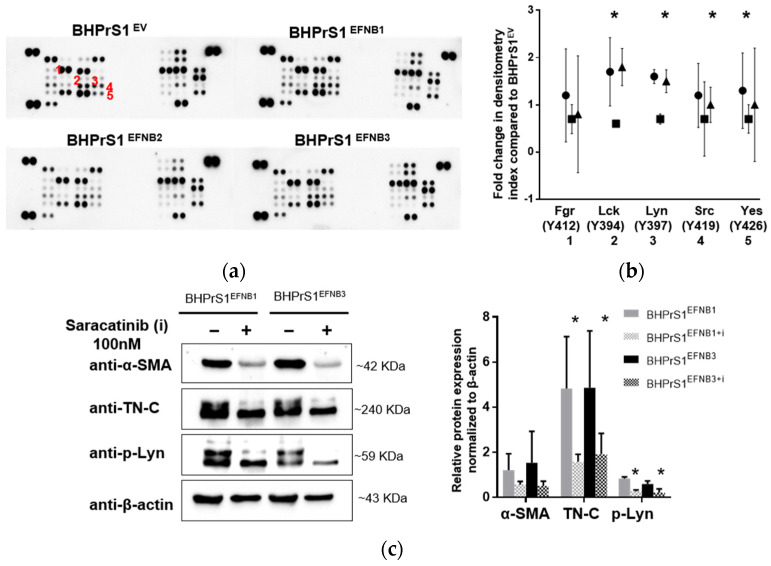
Ephrin ligands expression activates Src family kinases (SFK) in stromal cells. (**a**) Phospho-kinase array in control and EFNB ligands overexpressed BHPrS1 cells revealed overexpression of Src family kinases (SFK) in BHPrS1^EFNB1^ and BHPrS1^EFNB3^ compared to BHPrS1^EV^.Densitometry analysis of SFK phospho-protein arrays show marked phosphorylation of SFK family members Fgr (1), Lck (2), Lyn (3), Src (4) and Yes (5). (**b**). Data are presented as fold changes of BHPrS1^EFNB1^ (circles), BHPrS1^EFNB2^ (squares), and BHPrS1^EFNB3^ (triangles) compared to controls (BHPrS1^EV^) (tr = 2, * *p* < 0.05). (**c**) Both BHPrS1^EFNB1^ and BHPrS1^EFNB3^ were treated with Saracatinib (100 nM) for 48 h to inhibit SFK activation. After Saracatinib exposure, α-SMA (alpha smooth muscle actin) and TN-C (Tenascin C) expression in BHPrS1^EFNB1^ and BHPrS1^EFNB3^ cell lines went down (Left). *p*-Lyn, α-SMA and TN-C expressions were quantified and normalized to β-actin (Right). Data is presented as mean of three independent biological experiments (* *p* < 0.05).

To investigate downstream signaling of EFNB ligands overexpression in BHPrS1 cells, we quantified the expression of a panel of phospho-kinases using an antibody-based array in BHPrS1^EV^, BHPrS1^EFNB1^, BHPrS1^EFNB2^, and BHPrS1^EFNB3^ cells. Quantitation of the intensity demonstrated that phosphorylation signals were differentially activated in each of the EFNB-expressing cell lines compared to BHPrS1^EV^ (Figure 6a and Figure S4). Notably, overexpression of EFNB ligands exhibited changes in tyrosine phosphorylation of several members of the Src family kinases (SFKs) including Src, Lck, Lyn, and Yes (Figure 6a,b) compared to BHPrS1^EV^. BHPrS1^EFNB1^ showed high phosphorylation in Src family kinases Lck (Y394), Lyn (Y397), Yes (Y426), and Src (Y419) (Figure 6b) compared to BHPrS1^EV^. In contrast, BHPrS1^EFNB2^ displayed significantly lower amounts of phosphorylation in SFKs compared to BHPrS1^EV^. Among all the SFKs, BHPrS1^EFNB3^ had significantly higher phosphorylation of only Lck and Lyn at Y394 and Y397 sites (Figure 6b). These results show that SFK phosphorylation can be activated (in EFNB1 and EFNB3) or suppressed (EFNB2) by EFNB ligands in prostate fibroblasts. Others have shown the potential role of SFKs in the activation of fibroblasts in different organs and their promotion of fibrosis [43,44,45,46]. Next, we studied whether SFK phosphorylation is required for EFNB-induced CAF activation.

### 3.8. Inhibition of SFK Suppressed CAF Activation In Vitro

SFKs are involved in the pathogenesis of several cancers, including PCa [47,48,49], and activation of fibroblasts in different organs [43,44,45,46]. The Src inhibitor Saracatinib (AZD0530) has been shown to have antitumor activities in cancer cells and is currently being evaluated in a Phase 1b/2a clinical trial for the treatment of Idiopathic Pulmonary Fibrosis (NCT04598919). Both in vitro and in vivo studies show that inhibition of SFK blocks the activation of fibroblasts [43]. We used Saracatinib (AZD0530) to block Src family kinases to determine whether SFK phosphorylation is involved in CAF activation in EFNB1- and EFNB3- expressing BHPrS1 cells. We inhibited SFK using Saracatinib at 100 nM and evaluated the phosphorylation of Src (Y419), Lck (Y394), and Lyn (Y397). We tested different time points for Saracatinib-induced inhibition of SFK phosphorylation and found decreased phosphorylation (Src Y419, Lck Y394, and Lyn Y397) levels after 60 min (Figure S5a,b). After 48 h of treatment with Saracatinib, we isolated protein from BHPrS1^EFNB1^ and BHPrS1^EFNB3^ cells and assessed the expression of CAF markers. At 100 nM concentration, Saracatinib effectively reduced the expression of α-SMA and TN-C in both BHPrS1^EFNB1^ and BHPrS1^EFNB3^ fibroblasts (Figure 6c). A slight decrease in α-SMA expression was noted in BHPrS1^EV^ cells upon saracatinib inhibition (Figure S5c). Taken together, these data points suggest that Saracatinib blocked the activation of SFK and prevented EFNB1/3-induced expression of CAF markers ɑ-SMA and TN-C in vitro.

## 4. Discussion

Stromal remodeling in the TME can be found from the initial stages of PCa and throughout the progression of the tumors [42]. Genetic alterations in cancer cells, such as loss of tumor suppressors and/or accumulation of oncogenes, result in phenotypic alterations that can activate neighboring fibroblasts to acquire CAF activity [50,51]. Despite EphB2′s potential tumor suppressor effect in PCa cells [21,28], little is known about the role of the cognate ligands, EFNBs, in the TME. This is the first study to report the functional consequences of EFNB ligand overexpression in fibroblasts on PCa tumorigenesis. EFNB ligands were elevated in human prostate fibroblasts isolated from cancer (PZ) versus benign (TZ) tissue. Our results also indicate that transcripts encoding all three EFNB ligands are co-expressed. Both EFNB1 and EFNB3 expression correlated with EFNB2 levels. Interestingly, EFNB2 expression increased in the human prostate fibroblasts BHPrS1 when either EFNB1 or EFNB3 were overexpressed (Figure S6). It is not known whether a positive (or negative) feedback loop exists in the regulation of these EFNB paralogs. Recent studies have shown that the expression pattern of paralog genes and transcription factors could be clinically relevant [52]. Environmental stress could elicit the differential expression of paralog genes due to divergence of sequence or *cis*-regulatory elements [53,54]. The mechanism of EFNB ligand divergence needs to be determined. In addition to paralog regulation, our findings suggest that the heterogeneous expression of EFNB ligands between patients in primary human prostate fibroblasts may reflect their intrinsic heterogeneity, and more investigation is needed to better identify the role of EFNB-expressing CAFs clusters in PCa tumorigenesis.

Given the complexity of sub-populations in CAFs, the search for a gene or a set of genes to be used for their identification has been challenging. To date, there is no single marker that identifies or separates CAF populations from other cell types [55,56,57]. However, there are a wide variety of genes reported to be enriched in fibroblasts with CAF characteristics. Some of the well-accepted markers include α-SMA, TN-C, fibroblast specific protein 1 (FSP-1), fibroblast activated protein (FAP), PDGFR-α, PDGFR-β, Thy-1, Podoplanin, Integrin β1, and Caveolin-1 [56,58]. In this study, we showed that overexpression of EFNB1 and EFNB3 in a normal prostate fibroblast line (BHPrS1) increased the expression of the myofibroblast-associated gene TN-C both in vitro and in vivo. TN-C, an ECM protein, is found in abundance in both cancer and stromal cells [59] and is utilized as a biomarker to assess cancer progression and therapy response [60]. PCa lymph node metastases are linked to higher levels of TN-C in the prostate fibroblasts [61]. TN-C is also reported to play an important role in cancer cell proliferation, migration, and invasion [59]. TN-C signaling from stromal cells may have an impact on the cancer cells’ ability to invade [62]. Other Ephrin ligands, such as Ephrin A5, have a role in fibroblast activation. An in vitro study of murine fibroblasts and CAFs isolated from pancreatic ductal adenocarcinoma has shown the upregulation of different types of collagen by the EFNA5 ligand [63,64]. In this study we found that EFNB1 and EFNB3 overexpressing fibroblasts induced in vivo collagen deposition and TN-C expression, creating a favorable environment to foster PCa tumorigenicity.

Stromal cells influence PCa cell biological behavior via paracrine activation pathways. We observed enhanced in vitro BPH1 cell proliferation upon exposure to secreted factors that emanate from EFNB-expressing fibroblasts. We report for the first time that increased expression of EFNB ligands in normal prostate fibroblasts could activate them into CAF-like phenotypes. Genetically unstable BPH1 cells are considered to represent an initiated or premalignant prostatic epithelium, which can fully transform in vivo under oncogenic pressure exerted by CAF [12]. Stromal-epithelial interactions play an important role directly on cancer cell behavior and indirectly affect the TME, supporting neoangiogenesis and escape from immunosurveillance to induce tumor progression and metastasis in PCa [13,65]. We observed enhanced proliferation and tumorigenicity of BPH1 and LNCaP cells in the presence of fibroblasts with increased expression of EFNB1 or EFNB3 ligands. However, EFNB2 displayed tumor-suppressive effects, which were more pronounced in BPH1 cells. Tumor angiogenesis, one of the key hallmarks of cancer, promotes tumor growth and invasion. Tumor angiogenesis is increased by pro-angiogenic factors such as VEGF and inhibited by anti-angiogenic factors such as TSP-1. The net ratio of factors inhibiting or promoting angiogenesis can be used clinically as therapy response predictors [66]. Increases in the ratio of TSP-1 to VEGF secretions were identified in EFNB2 overexpressing fibroblasts, suggesting increased anti-angiogenic effects that could partially explain the reduced tumorigenicity. In contrast, BHPrS1^EFNB1^ and BHPrS1^EFNB3^ expressed elevated VEGF. In both LnCAP and BPH1, increased cell proliferation (Ki67), and collagen deposition (picrosirius red) were found in recombinant grafts with EFNB1 and EFNB3 fibroblasts. These findings suggest a novel role for stromal EFN-B ligands in relation to CAF activation and TME remodeling that can support tumor progression.

Several well-established activation signals in CAFs include modulation of TGFß signaling through the SMAD transcription factors, Notch signaling, NF-κB acting on signal transducer and activator of transcription (STAT) transcription factor, SRF-driven transcription, and Yes-associated protein 1 (YAP1)-TEAD-driven transcription [9,67,68,69]. Analyzing the phosphorylation profiles of kinases revealed that overexpression of EFNB1 and EFNB3 ligands resulted in enhanced phosphorylation of SFKs, while EFNB2-expressing fibroblasts resulted in diminished phosphorylation of SFKs. SFKs have been shown to be involved in the regulation of cytoskeletal architecture as well as the development of integrin-dependent signaling responses in fibroblasts [70,71,72,73]. Increased SFK activity is associated with pathogenesis of several diseases, including PCa [47]. In PCa cells, activation of Src increases proliferation and migration by regulating cyclin D1 and c-Myc [47]. Src inhibition blocks the ERK 1/2 and Akt signaling pathways, resulting in decreased PCa cell proliferation [47]. Loss of SFKs causes VEGF downregulation in colon cancer cells [48]. In our study, we found downregulation of SFKs and VEGF in EFNB2-expressing fibroblasts, whereas EFNB3 overexpressed fibroblasts had higher secretions of VEGF and phosphorylation of Lck and Lyn. Activation of SFKs can drive renal interstitial fibroblast activation and further inhibit Src-induced fibrosis [44]. Inhibition of another SFK, Fyn, was found to have potential clinical utility by inhibiting fibroblast activation associated with liver fibroblasts [43]. Aligned with these findings, we report for the first time that SFKs may be involved in activation of prostate stromal cells by modulating α-SMA and TN-C in response to increased EFNB1 and EFNB3 expression. Further studies are needed to better delineate the mechanisms involved in SFK activation by EFNB ligands in prostate fibroblasts.

The role of Ephrins in the biology of CAFs and their impact on tumor growth has received limited attention [64,74]. A plethora of studies have implicated EFNB ligands in cancer cell biology, and several studies report paradoxical effects of EFNB1, EFNB2, and EFNB3 ligands. EFNB1 expression is high in cancer cells and is potentially associated with tumorigenesis of gastric [75], ovarian [76], and brain cancer [77]. Some reports suggest the use of EFNB1 as a potential biomarker for tumor progression and therapy response in the brain [77] and breast cancer [78]. Similarly, EFNB2 and EFNB3 are reported to be highly expressed in different types of cancer cells and are associated with poor prognosis [79,80,81]. In contrast, a recent study in breast cancer cells reported that higher expression of EFNB2 may be associated with delayed metastasis and better prognosis by reducing cell proliferation, migration, and invasion [82]. In this study we showed the paracrine effects of EFNB ligand activation through the modulation of the fibroblast secretome. However, it is important to note that EFNB ligands can bind to different Eph receptors in a cell-to-cell contact fashion. Therefore, we cannot exclude the possibility of forward signaling in cancer cells in our in vivo studies. BPH1 cells express more EphB2 receptors compared to LNCaP cells [83]. It is possible that epithelial cells expressing EFNB cognate receptors for EFNB ligands may actively respond to stromal signals via forward signaling, thereby promoting the motility and invasion observed in vivo. Forward signaling between fibroblasts and/or autocrine signaling is another possibility. Increasing the expression of EFNB ligands did not alter the expression of EphB2; however, the expression of the EphB4 receptor was reduced in EFNB2-overexpressing fibroblasts (Figure S7). EphB4 in cancer cells has paradoxical effects depending on whether or not its cognate ligand EFNB2 is present. In the presence of its cognate ligand, EFNB2, it can act as a tumor suppressor. However, in the absence of the ligand, the receptor is tumor-promoting [30]. Whether EphB4-EFNB2 interactions in CAFs represent a similar situation observed in cancer cells is currently unknown. In addition to paracrine induced effects upon stromal overexpression of EFNB ligands, further studies on receptor-ligand crosstalk between stromal-epithelial interactions and autocrine signaling are warranted.

## 5. Conclusions

The present study shows several novel functions of Ephrin B signaling activation in prostate stromal cells and may have significant implications during tumor progression. In summary, our findings indicate that overexpression of prostate stromal EFNB ligands (EFNB1 and EFNB3) plays a role in PCa tumorigenesis by modulating the TME through alterations in the fibroblast secretome, with multiple effects on neovascularization, collagen deposition, cancer cell proliferation, and migration. The potential anti-tumorigenic and anti-angiogenic role of EFNB2 in prostate stromal cells requires further research. Whether targeting SFK is a valid approach to regulate the tumor-promoting effects in Ephrin-expressing tumors needs to be tested in preclinical models. Ephrin ligands are a relatively new and fast expanding area of cancer research. Our observations provide a foundation to explore their significant potential utility for cancer treatment.

## Data Availability

The data presented in this study are available on request from the corresponding author. The data are not publicly available due to privacy (General Data Protection Regulation, GDPR) and ethical restrictions.

## Supplementary Materials

The following supporting information can be downloaded at: https://www.mdpi.com/article/10.3390/cancers14092336/s1. Figure S1: Paracrine signals from Ephrin B engineered BHPrS1 cells effect on LNCaP cell proliferation; Figure S2: Differential secretion of cytokine proteins in the media of normal prostate fibroblasts (BHPrS1) expressing Ephrin B ligands; Figure S3: Increased stromal EFNB1 and EFNB3 induce LNCaP proliferation and TME remodeling in vivo; Figure S4: Differential protein expression of phosphorylation of a number of kinases in Ephrin B ligands expressing fibroblasts (BHPrS1); Figure S5: Differential expression of phosphor Src family kinases upon inhibition with 100nM Saracatinib; Figure S6: mRNA expression of EFNB2 in BHPrS1^EFNB1^, and BHPrS1^EFNB3^ cell lines; Figure S7: EphB2 and EphB4 expression in engineered BHPrS1 cell lines; Figure S8: Uncropped Western blots corresponding to Figure 2a; Figure S9: Uncropped Western blots corresponding to Figure 2b; Figure S10: Uncropped Western blots corresponding to Figure 6c; Figure S11: Uncropped Western blots corresponding to Figure S5a; Figure S12: Uncropped Western blots corresponding to Figure S5c.
