# Supplementary material for: Ephrin B Activate Src Family Kinases in Fibroblasts Inducing Stromal Remodeling in Prostate Cancer
Source: Cancers (Basel). 2022 May 9;14(9):2336. doi: 10.3390/cancers14092336 (PMC9102363; doi:10.3390/cancers14092336)
Supplement: Supplementary file 1 [file cancers-14-02336-s001.zip › cancers-1687376-supplementary.pdf]

cancers

Supplementary Materials: Ephrin B Activate Src Family Kinases in Fibroblasts Inducing Stromal Remodeling in Prostate Cancer

Mamatha Kakarla, Sathyavathi ChallaSivaKanaka, Mary F. Dufficy, Victoria Gil, Yana Filipovich, Renee Vickman, Susan E. Crawford, Simon W. Hayward and Omar E. Franco *

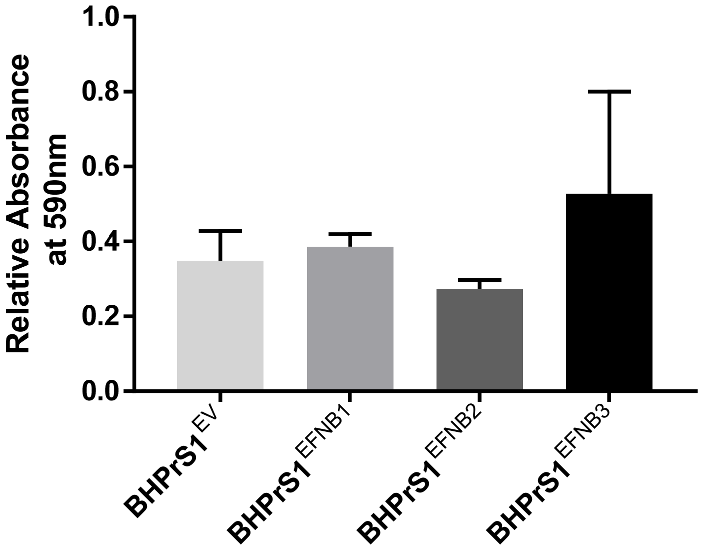

Figure S1. Paracrine signals from Ephrin B engineered BHPrS1 cells effect on LNCaP cell proliferation. LNCaP cells were cultured in the presence of conditioned media from EFNB ligand-expressing BHPrS1 ($BHPrS1^{EFNB1}$, $BHPrS1^{EFNB2}$, $BHPrS1^{EFNB3}$ and $BHPrS1^{EV}$). After 5 days of exposure, cells were stained with crystal violet and the absorbance at 590 nm quantified. Data is presented as mean ± SEM (n=5 independent experiments, p>0.05, one-way ANOVA).

cancers

MDPI

S2a

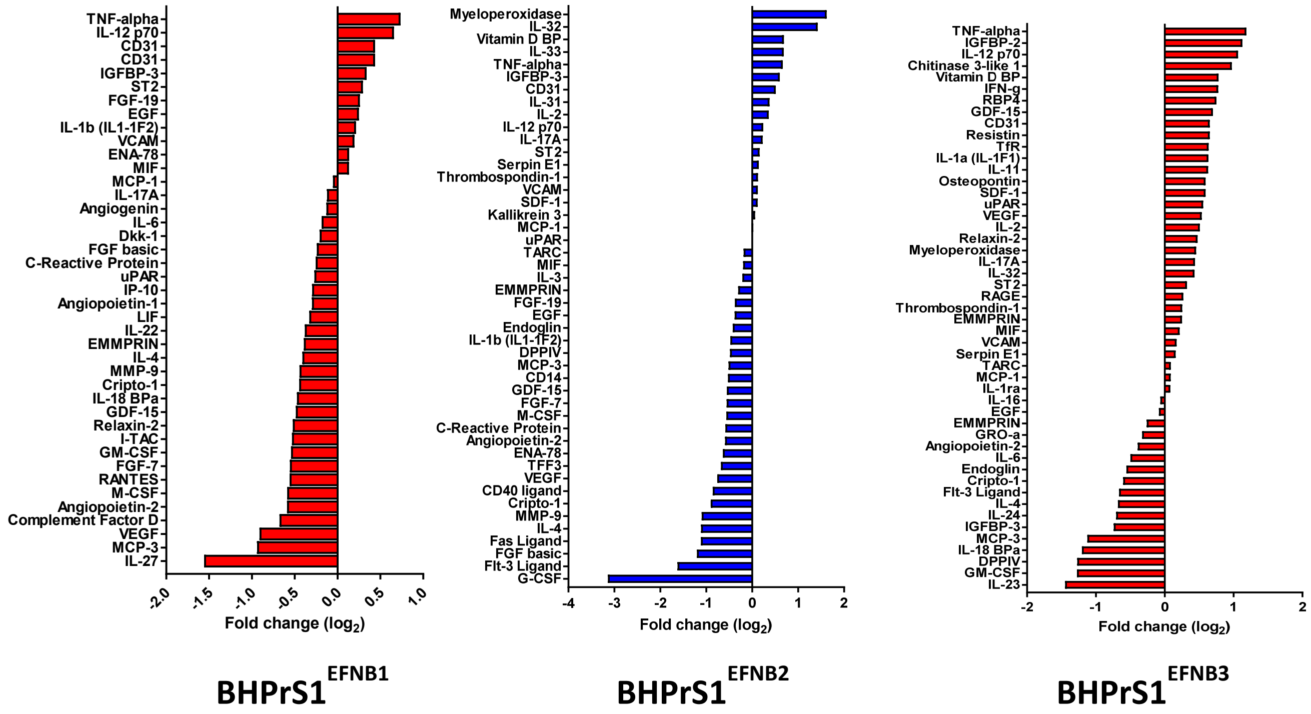

S2b

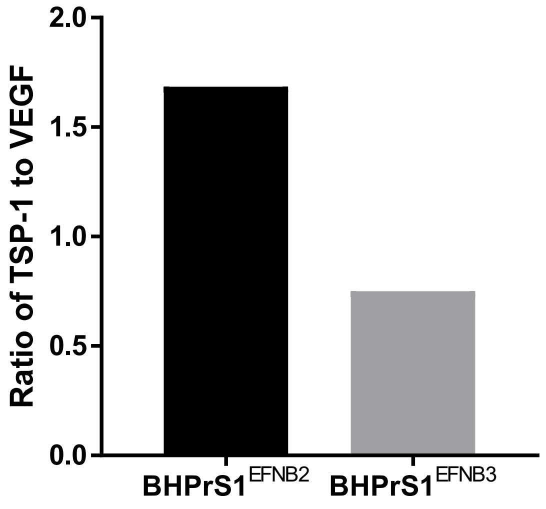

Figure S2. Differential secretion of cytokine proteins in the media of normal prostate fibroblasts (BHPrS1) expressing Ephrin B ligands. a) Log graphs of $BHPrS1^{EFNB1}$, $BHPrS1^{EFNB2}$, $BHPrS1^{EFNB3}$ based on the fold change of cytokines that are significantly different compared to $BHPrS1^{EV}$ in the cytokine array (technical replicates =2). b) Ratio of TSP-1 to VEGF is higher in $BHPrS1^{EFNB2}$ compared to $BHPrS1^{EFNB3}$. TSP-1: Thrombospondin-1; VEGF: Vascular endothelial growth factor.

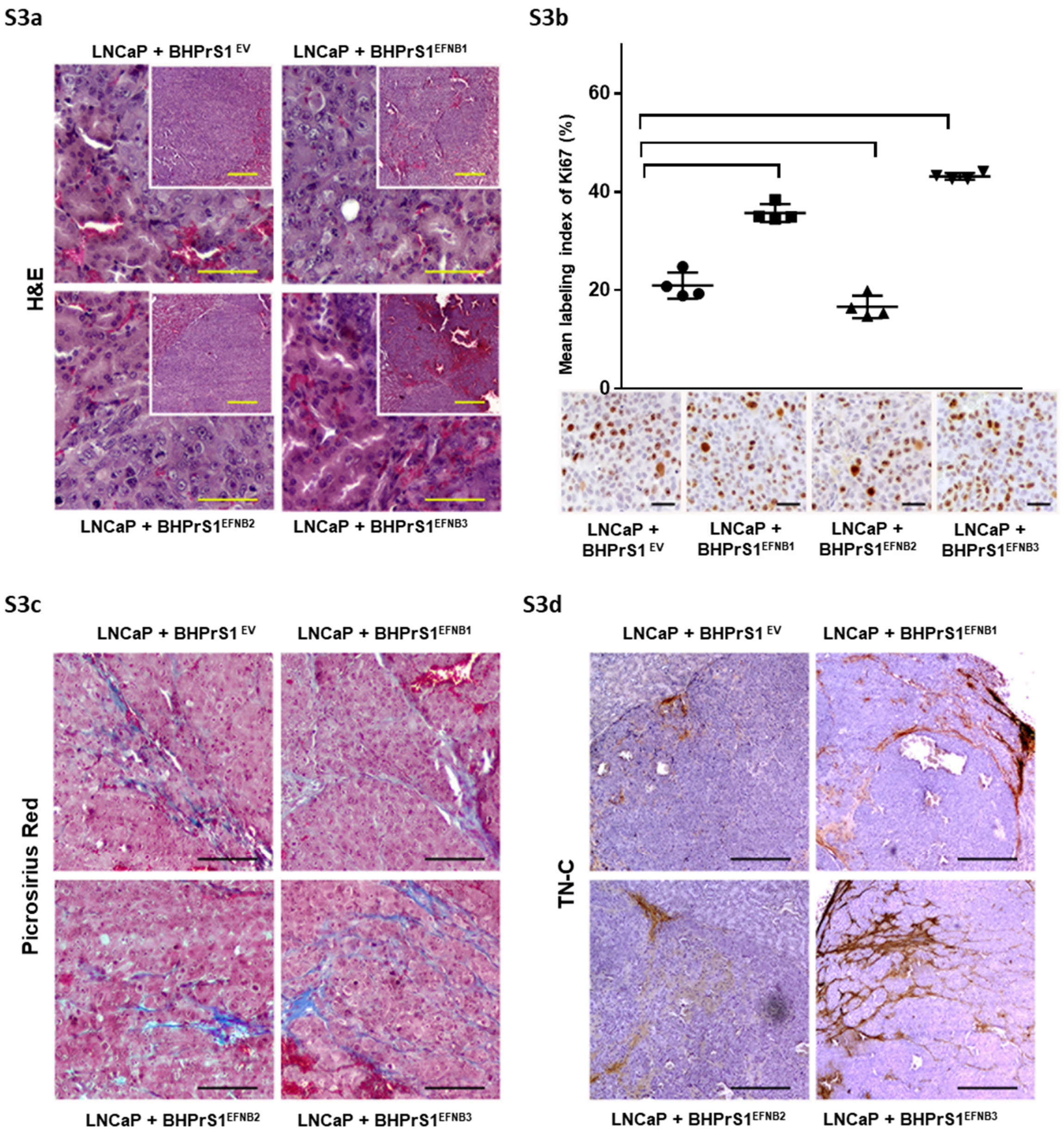

Figure S3. Increased stromal EFNB1 and EFNB3 induce LNCaP proliferation and TME remodeling in vivo.

Hematoxylin and eosin (H&E) stained sections of the tumors resulting from grafts of LNCaP with engineered BHPrS1 cell lines. Grafts of LNCaP with BHPrS1^{EFNB1} and BHPrS1^{EFNB3} showing pronounced inflammatory infiltrates compared to BHPrS1^{EV}. (b) Immunohistochemical (IHC) staining showing pronounced Ki67 expression in LNCaP tumors with BHPrS1^{EFNB1} and BHPrS1^{EFNB3} and reduced Ki67 expression in BHPrS1^{EFNB2} compared to BHPrS1^{EV}. Dot plot showing Ki67 expression quantification (one-way ANOVA). (c) Higher collagen deposition in LNCaP tumor grafts with BHPrS1^{EFNB1} and BHPrS1^{EFNB3} compared to BHPrS1^{EV} as shown by picrosirius red staining (d) ECM remodeling marker tenascin-C (TN-C) is highly expressed in tumor grafts of LNCaP with BHPrS1^{EFNB1} and BHPrS1^{EFNB3} compared to BHPrS1^{EV}. Grafts of LNCaP with BHPrS1^{EFNB2} and BHPrS1^{EV} has relatively lower expressions of TN-C. Scale bar in yellow and black lines represents all pictures were taken at same magnification.

cancers

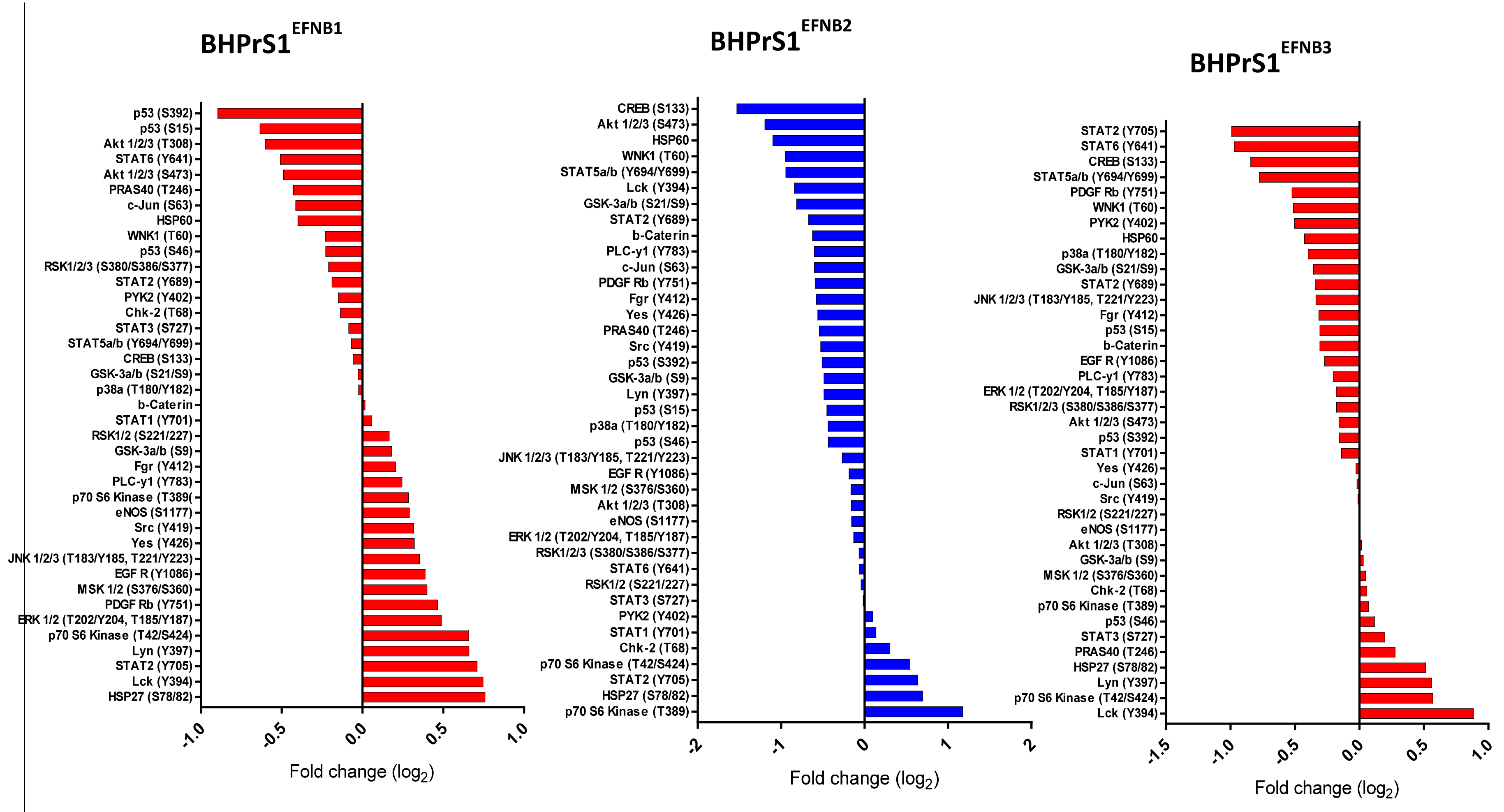

Figure S4. Differential protein expression of phosphorylation of a number of kinases in Ephrin B ligands expressing fibroblasts (BHPrS1). Log graphs of BHPrS1^{EFNB1}, BHPrS1^{EFNB2}, BHPrS1^{EFNB3} phospho kinases based on the fold change of 2 technical replicates compared to BHPrS1^{EV}.

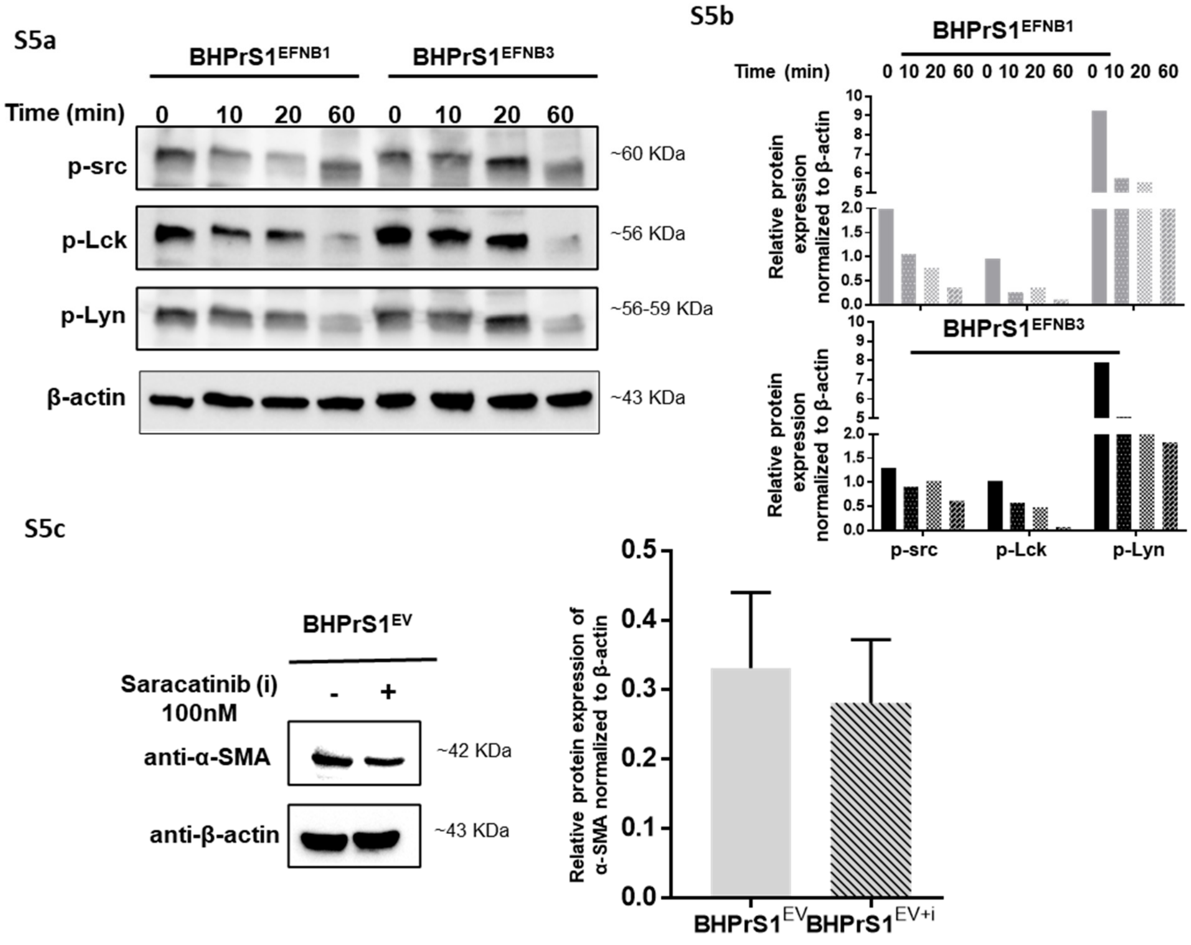

Figure S5. Differential expression of phosphor Src family kinases upon inhibition with 100nM Saracatinib. S5a. Western blot of BHPrS1EFNB1, and BHPrS1EFNB3 showing reduction in phosphorylation of src, lck and lyn after treatment of saracatinib (100nM) for 0 min, 10 min, 20 min and 60 min (Left). S5b. p-src, p-lck, and p-lyn were quantified and normalized to β-actin and presented as the mean (Right) of one biological experiment. S5c. The protein levels of Alpha-smooth muscle actin (α-SMA) were evaluated in BHPrS1EV, and BHPrS1EV treated with saracatinib inhibitor (100nM) and normalized to β-actin. Data is presented as mean of two independent biological experiments and the error bars represent standard deviation.

cancers

MDPI

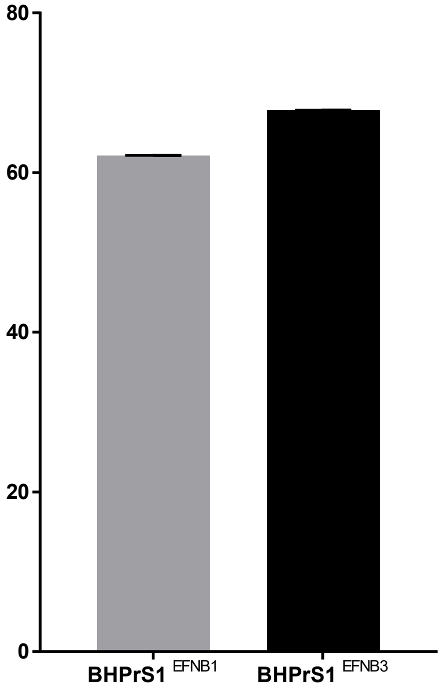

Figure S6. mRNA expression of EFNB2 in BHPrS1[EFNB1], and BHPrS1[EFNB3] cell lines. Values are representative of 3 independent experiments and data is presented as mean ± SEM.

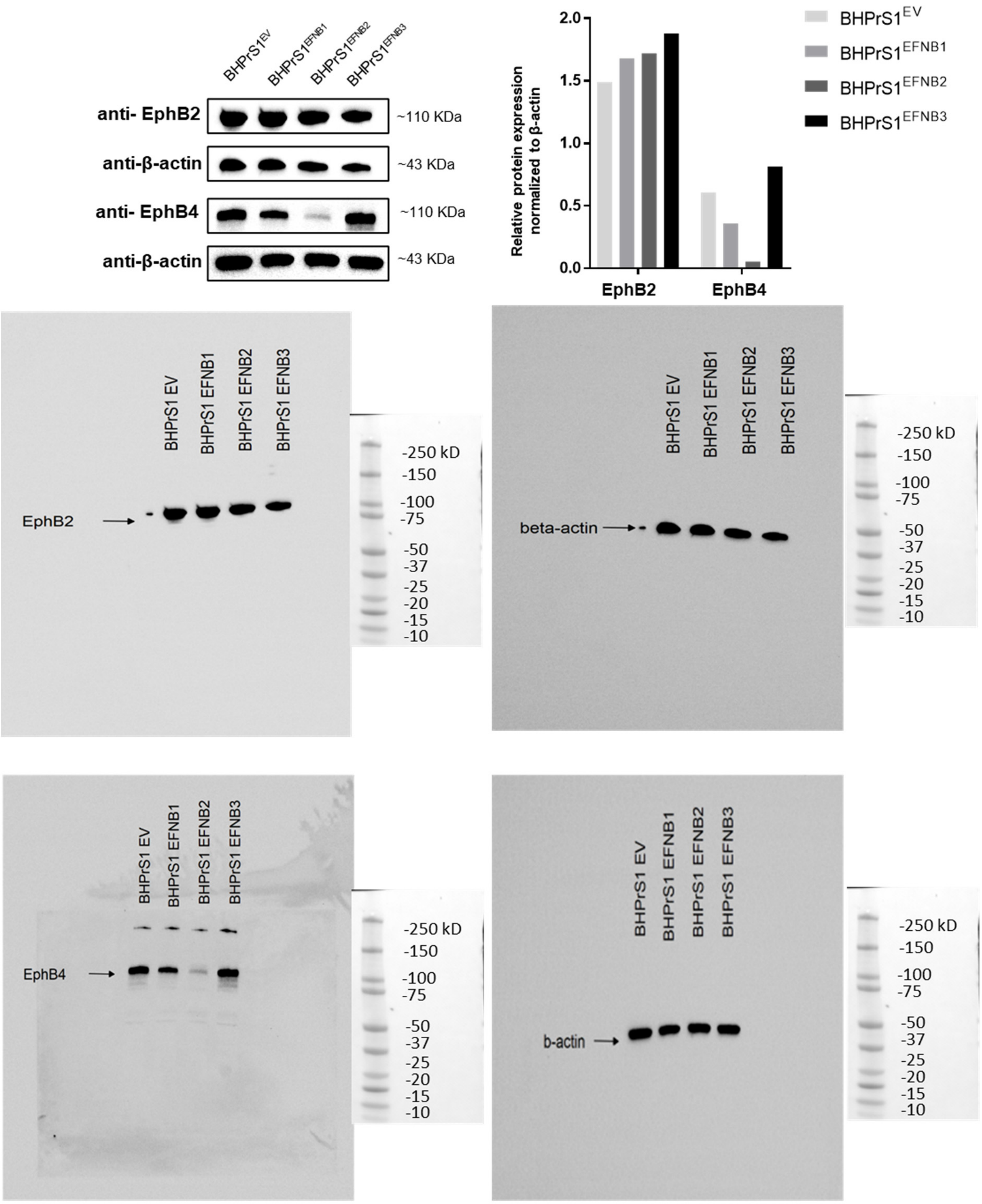

Figure S7. EphB2 and EphB4 expression in engineered BHPrS1 cell lines. The protein levels of Ephrin receptors – EphB2 and EphB4 were evaluated in Ephrin-generated cell lines (BHPrS1EFNB1, BHPrS1EFNB2, BHPrS1EFNB3) by western blot (Left). The bands were quantified and normalized to β-actin and presented as the mean of one biological experiment (Right). Corresponding original western blots were shown below.

cancers

MDPI

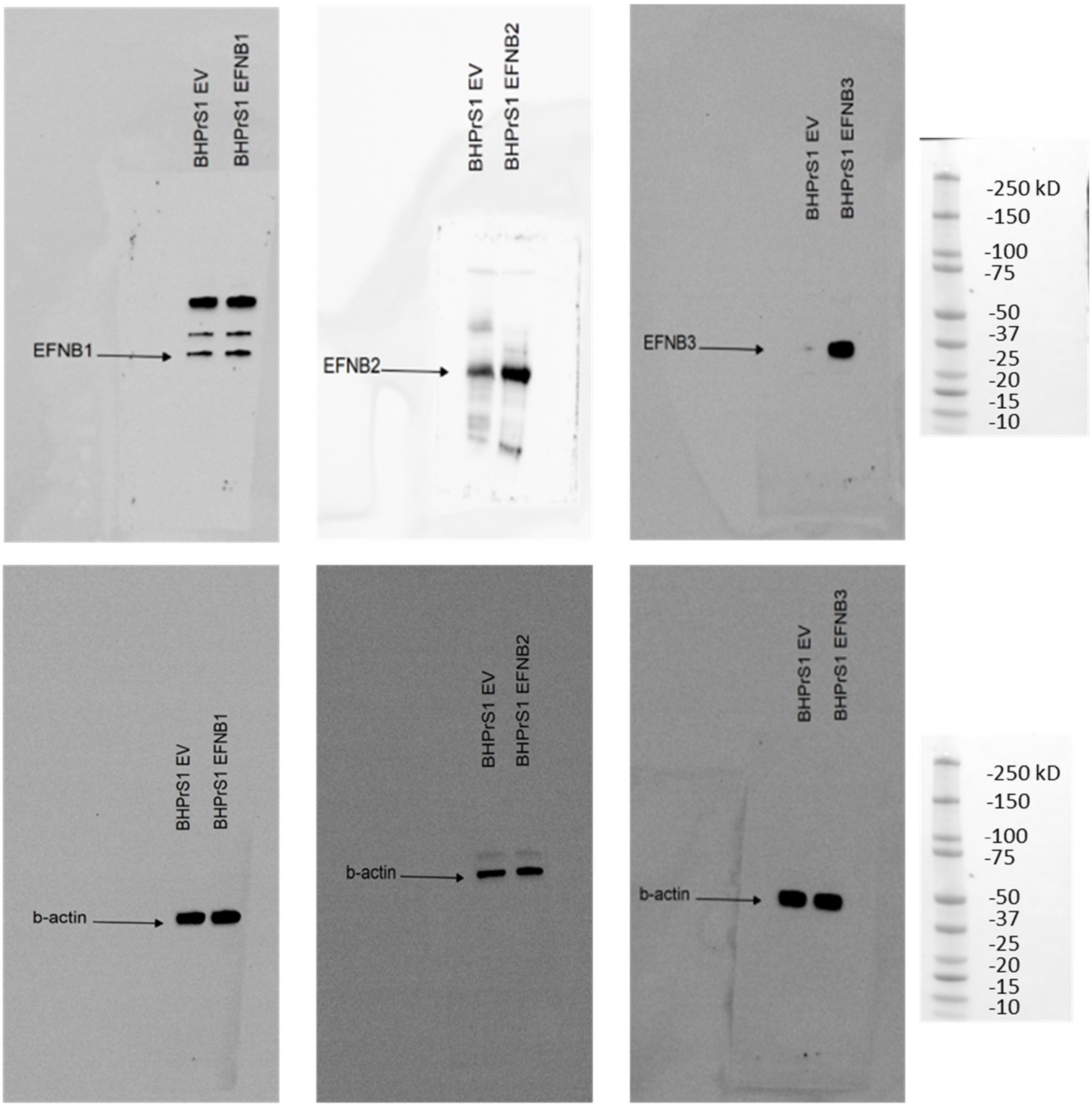

Figure S8. Uncropped western blots corresponding to Figure 2a and relative band intensities were shown.

cancers

MDPI

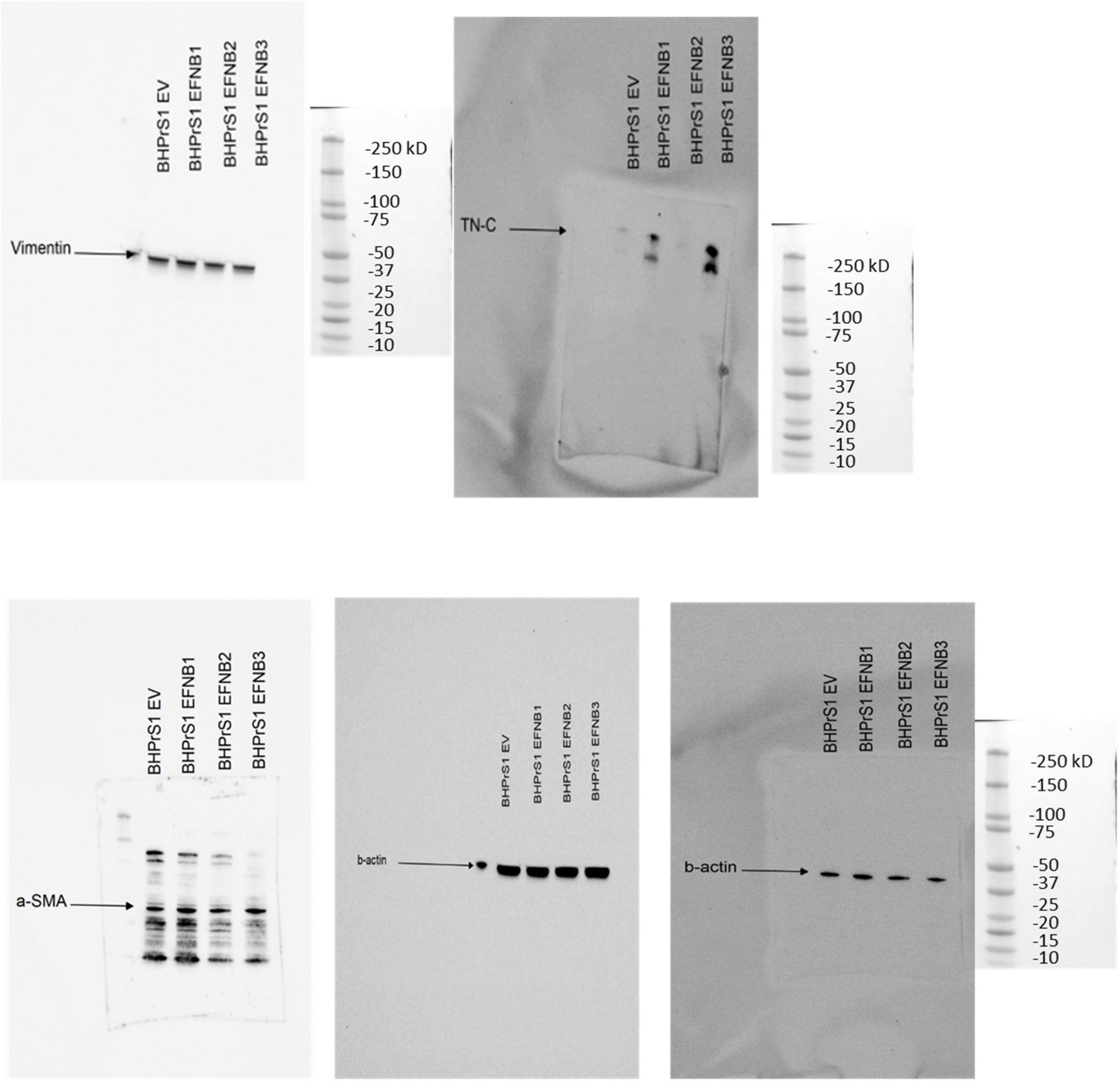

Figure S9. Uncropped western blots corresponding to Figure 2b and relative band intensities were shown.

cancers

MDPI

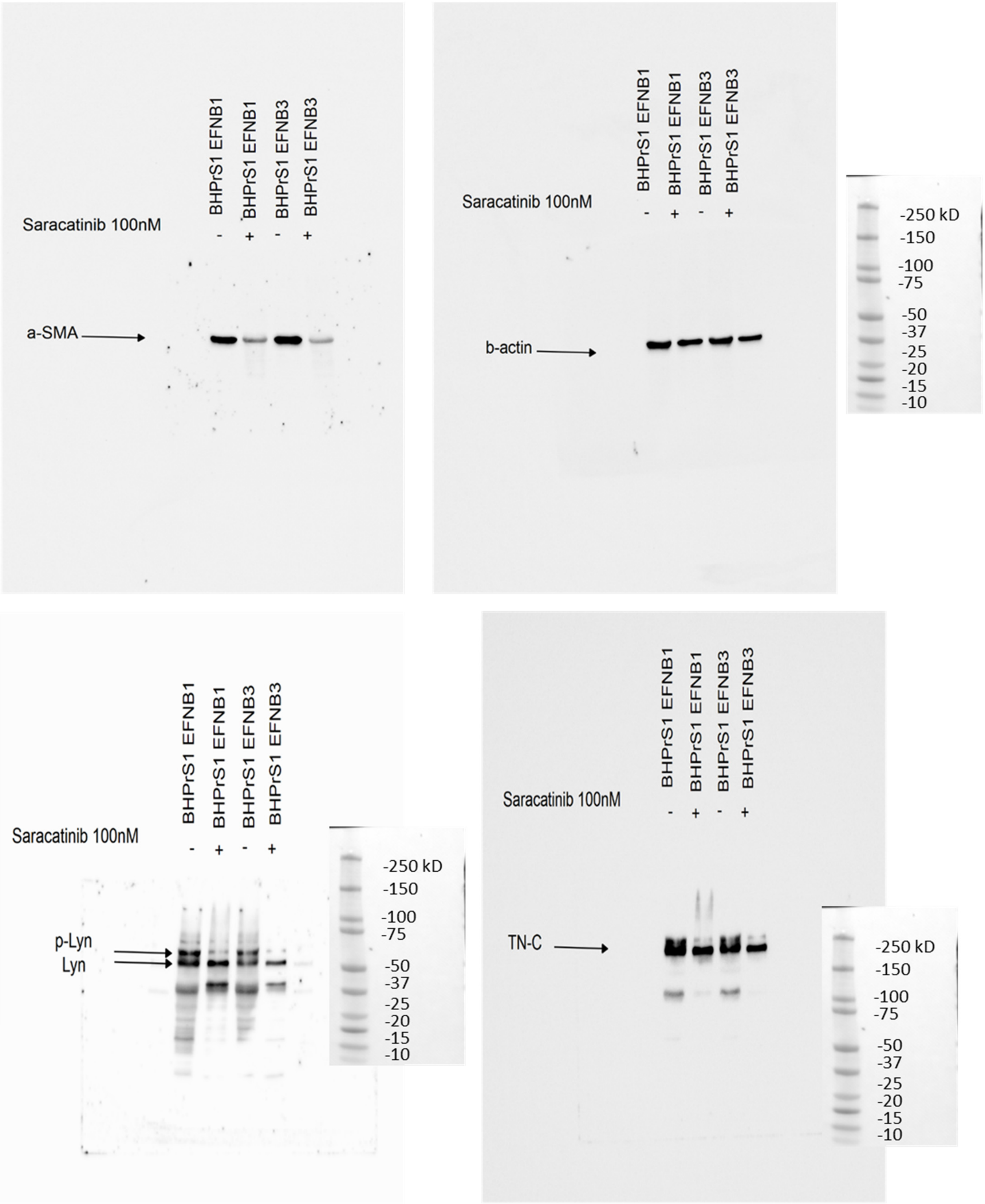

Figure S10. Uncropped western blots corresponding to Figure 6c and relative band intensities were shown.

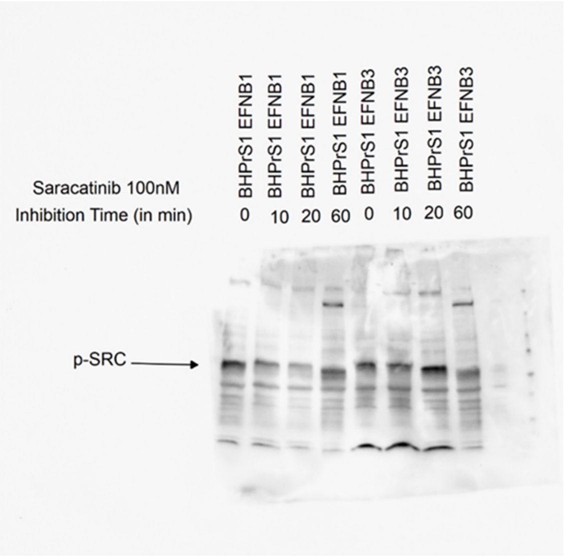
Saracatinib 100nM
Inhibition Time (in min)
BHPrS1 EFNB1
BHPrS1 EFNB1
BHPrS1 EFNB1
BHPrS1 EFNB1
BHPrS1 EFNB3
BHPrS1 EFNB3
BHPrS1 EFNB3
BHPrS1 EFNB3
0 10 20 60 0 10 20 60
p-SRC

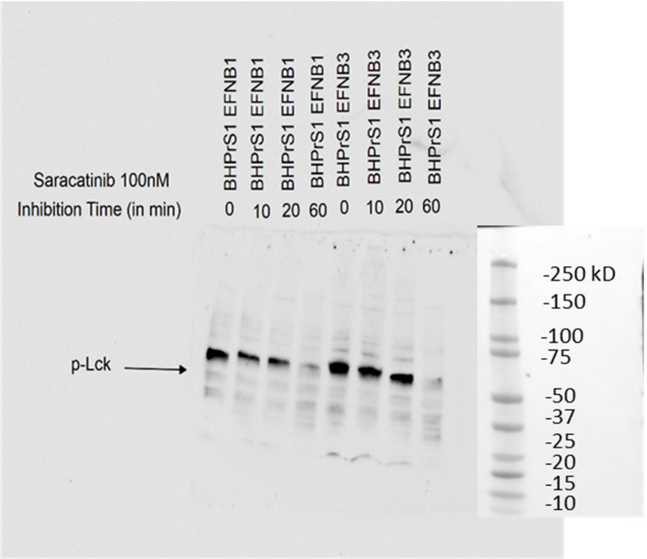
Saracatinib 100nM
Inhibition Time (in min)
BHPrS1 EFNB1
BHPrS1 EFNB1
BHPrS1 EFNB1
BHPrS1 EFNB1
BHPrS1 EFNB3
BHPrS1 EFNB3
BHPrS1 EFNB3
BHPrS1 EFNB3
0 10 20 60 0 10 20 60
p-Lck
-250 kD
-150
-100
-75
-50
-37
-25
-20
-15
-10

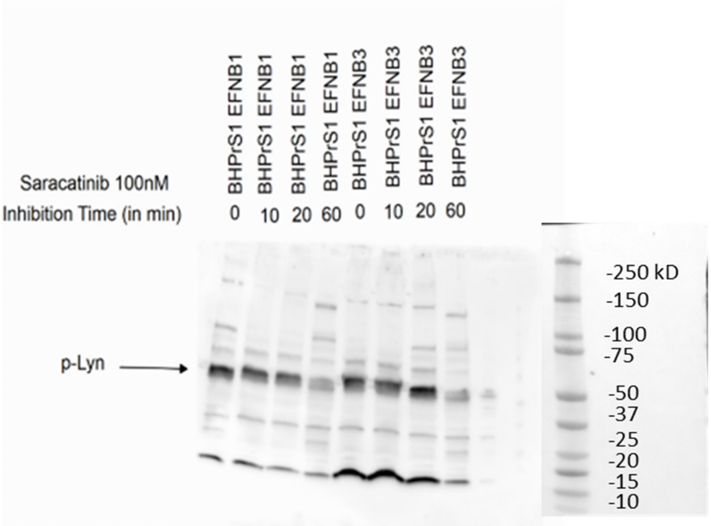
Saracatinib 100nM
Inhibition Time (in min)
BHPrS1 EFNB1
BHPrS1 EFNB1
BHPrS1 EFNB1
BHPrS1 EFNB1
BHPrS1 EFNB3
BHPrS1 EFNB3
BHPrS1 EFNB3
BHPrS1 EFNB3
0 10 20 60 0 10 20 60
p-Lyn
-250 kD
-150
-100
-75
-50
-37
-25
-20
-15
-10

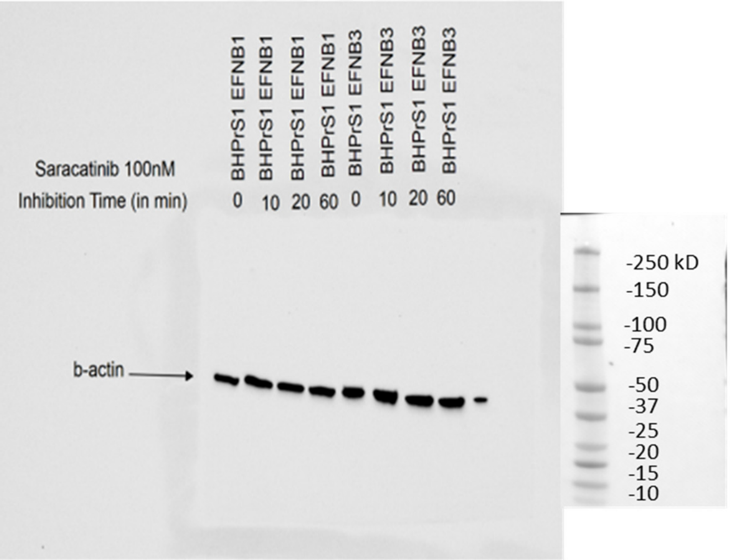
Saracatinib 100nM
Inhibition Time (in min)
BHPrS1 EFNB1
BHPrS1 EFNB1
BHPrS1 EFNB1
BHPrS1 EFNB1
BHPrS1 EFNB3
BHPrS1 EFNB3
BHPrS1 EFNB3
BHPrS1 EFNB3
0 10 20 60 0 10 20 60
b-actin
-250 kD
-150
-100
-75
-50
-37
-25
-20
-15
-10

cancers

Figure S11. Uncropped western blots corresponding to Figure S5a and relative band intensities were shown.

cancers

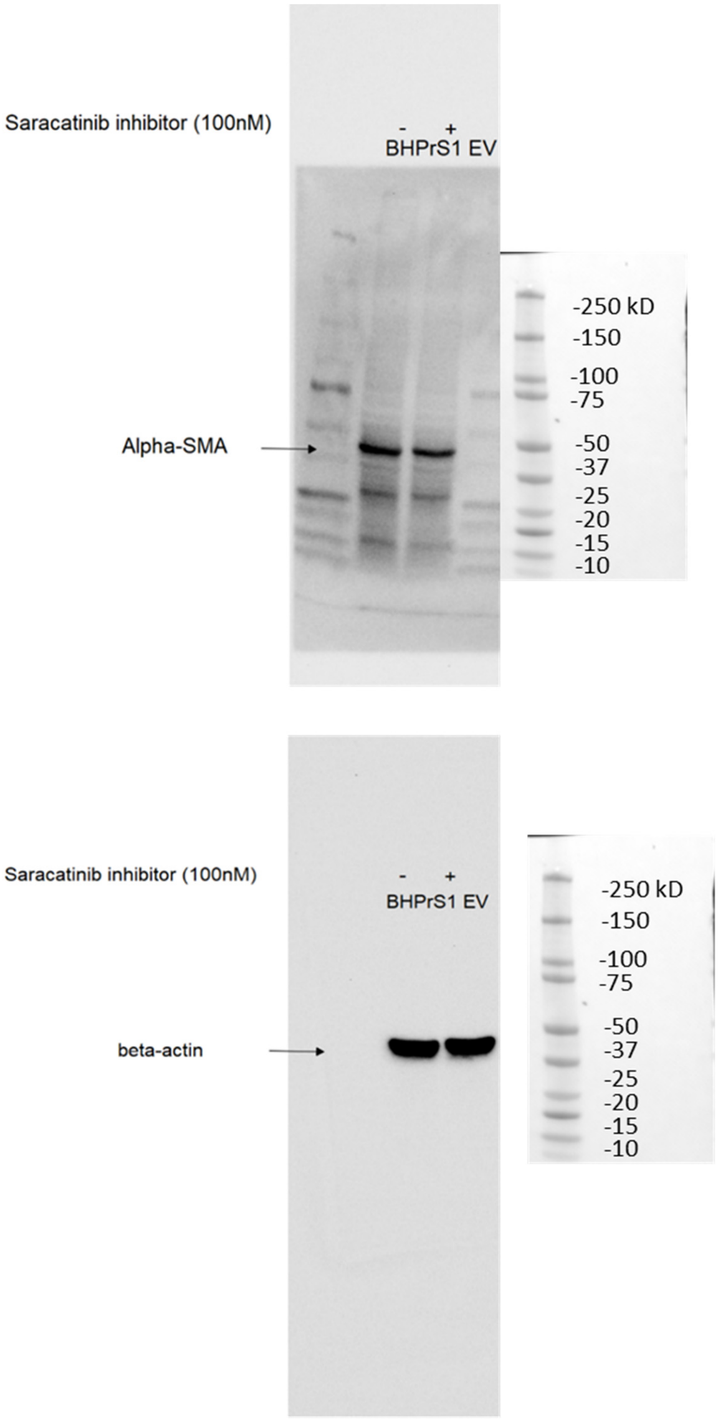

Figure S12. Uncropped western blots corresponding to Figure S5c and relative band intensities were shown.
